# Photonic‐Enabled Energy‐Efficient Transparent Neuromorphic Computing Devices: A Review

**DOI:** 10.1002/advs.75861

**Published:** 2026-06-01

**Authors:** Shuvaraj Ghosh, Ki‐bum Lee, Junghyeon Lee, Seunghee Cho, Malkeshkumar Patel, Hangfei Li, Yu Wen, Ye Zhou, Joondong Kim

**Affiliations:** ^1^ Photoelectric and Energy Device Application Lab (PEDAL), Multidisciplinary Core Institute For Future Energies (MCIFE) Incheon National University Incheon Republic of Korea; ^2^ Department of Electrical Engineering Incheon National University Incheon Republic of Korea; ^3^ State Key Laboratory of Radio Frequency Heterogeneous Integration Shenzhen University Shenzhen P. R. China; ^4^ Institute For Advanced Study Shenzhen University Shenzhen P. R. China

**Keywords:** artificial intelligence (AI), bionics, brain‐inspired computing, neuromorphic computing, photonic, transparent device

## Abstract

In the evolving field of artificial intelligence (AI), two notable trends are emerging: the rapid growth of large AI model sizes and the surge of vast amounts of data. Moore's law emphasizes the need for alternative computing paradigms to meet the rising demand for computational power and address von Neumann model constraints. Nowadays, neuromorphic computing, inspired by the mechanisms and functionality of human brains, uses physical artificial neurons to do computations and is drawing widespread attention. Neuromorphic computing aims to emulate brain‐like information processing with co‐localized memory and logic, breaking the von Neumann bottleneck. In this regard, the integration of photonic materials in computing led to growth in photonic computing, where light is a fundamental source of energy and can also be utilized as a signal for neuromorphic computing. Photonic computing enables ultrafast artificial neural networks with sub‐nanosecond latencies and low heat dissipation. However, current neuromorphic technologies still struggle to achieve petascale speed and energy efficiency. Additionally, the general benefits of neuromorphic computing and AI can be realized in an optically transparent manner, broadening their applications in bionics and human interfaces. This review explores the suitability and design strategies for transparent photonic devices that create artificial interfaces mimicking natural functions.

## Graphical abstract

Transparent photonic neuromorphic computing devices merge optics and brain‐inspired computing to overcome von Neumann bottlenecks with ultrafast, low‐energy processing. By exploiting transparent oxides, 2D materials, phase‐change materials, and hybrid heterostructures, these platforms enable photonic synapses, memory, and logic for see‐through edge intelligence, advancing smart displays, wearables, augmented reality, and bionic human‐machine interfaces.

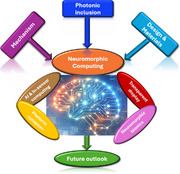

## 1 Introduction

**FIGURE 1:**
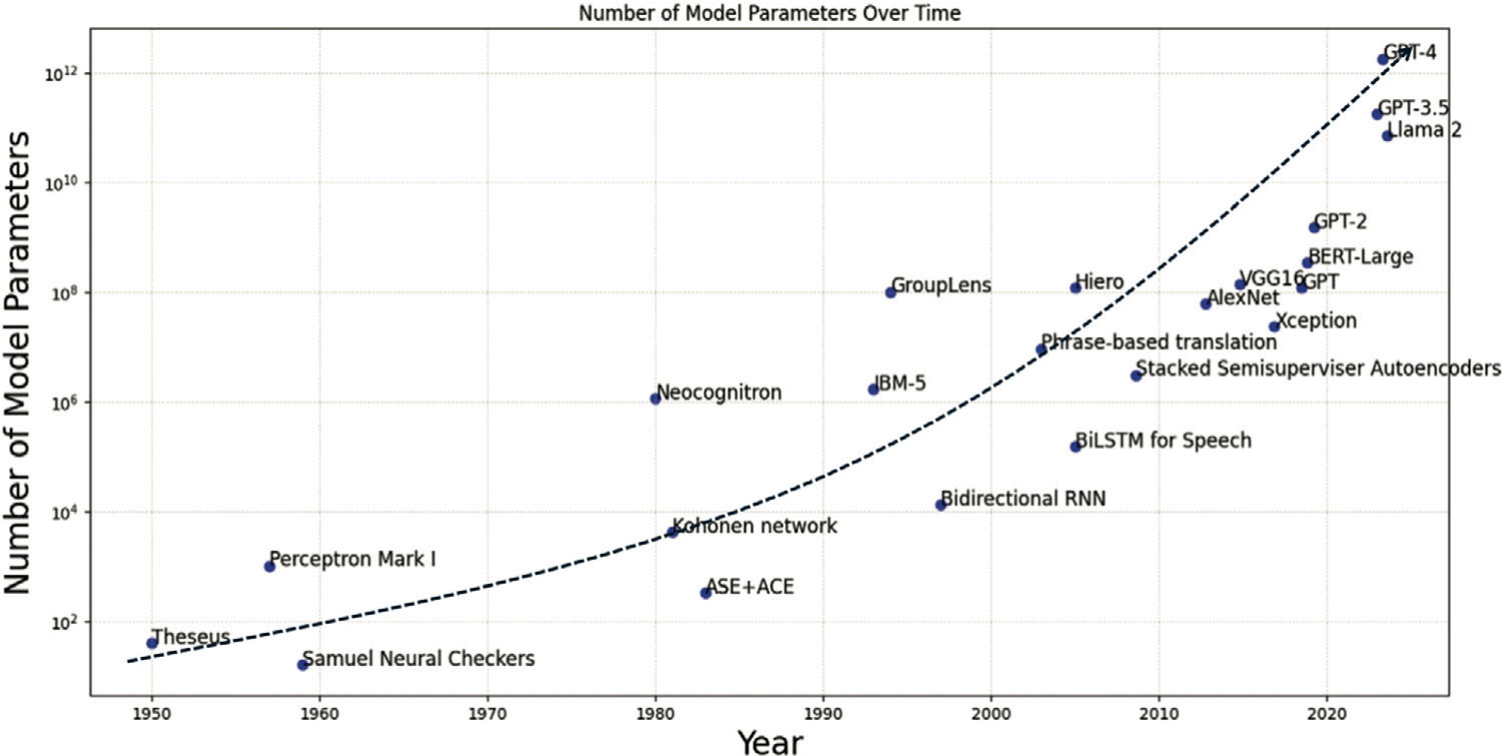
Trend of increase in representative AI model's parameters over time. Parameters (i.e., weights) are variables in an AI system whose values are adjusted during training to establish how input data is correlated with the output labels. The most recent ChatGPT has a record‐breaking 1.8 trillion parameters and is still growing. The number of parameters is estimated based on published statistics in respective papers and naturally comes with some uncertainty. Reproduced with permission [24]. Copyright 2025, Wiley‐VCH.

In recent years, there has been a substantial increase in the demand for high‐performance computers that can effectively support artificial intelligence (AI) applications. The number of programs utilizing deep learning training has increased twofold approximately every 3.5 months, a rate that significantly surpasses the performance doubling interval projected by Moore's law [1, 2]. Furthermore, it is essential that learning algorithms are capable of real‐time execution on the vast volumes of data generated by the numerous interconnected smart devices present within the Internet of Things (IoT) and edge computing environments. Figure 1 exhibits the trend that revolves around the expansion of AI model parameters. In Figure 1, a dashed trend line has been added to effectively depict the swift exponential increase in the number of parameters utilized in AI models. This visualization highlights the significant growth trend, emphasizing the expanding complexity and capability of modern AI systems. For instance, GPT‐4 (ChatGPT) is reported to have ≈ 1.8 trillion parameters, roughly an order of magnitude increase over GPT‐3 (175 billion). This supports the exponential increase shown in Figure 1. Nowadays, AI algorithms used in applications like autonomous vehicles (such as Tesla) and Amazon's Alexa are developed through neural networks (NNs), which are computational models inspired by the neuro‐synaptic architecture of the human brain. In this context, neuromorphic computing aims to emulate brain‐like architectures to achieve high‐speed, parallel processing with minimal energy. Conventional electronic hardware, such as central processing units (CPUs), graphics processing units (GPUs), and field‐programmable gate arrays (FPGAs), consumes on the order of 0.5 pJ in processing per multiply‐accumulate (MAC) operation, whereas the human brain performs ≈ 10^15^ operations at only ≈ 20 W [3, 4]. This immense efficiency motivates new hardware: for example, memristors can co‐locate memory and logic, alleviating the von Neumann bottleneck [5]. To address the challenge of the memory wall, an innovative strategy involves employing neuromorphic computing with memory devices such as memristors, which are designed to perform computations in situ where the data reside [6, 7, 8, 9, 10].

In the case of a single‐layer neural network, the total number of artificial synapses typically scales with the square of the number of artificial neurons. For instance, a fully connected network containing 10^4^ neurons necessitates 10^8^ synapses, which approaches the limitations imposed by current state‐of‐the‐art very‐large‐scale integration (VLSI) technology [5]. In this context, the integration of photonics with artificial neural networks provides significant benefits, such as parallel processing and extensive interconnectivity, which are essential for developing large‐scale photonic neuromorphic computing systems [11, 12, 13, 14]. Since photons can traverse three‐dimensional (3D) spaces without mutual interference, optical systems offer superior space‐bandwidth‐product performance and exceptionally rapid propagation speeds compared to electronic systems. These distinctive optical characteristics have generated significant interest in the development of photonic artificial NNs capable of executing neuromorphic functions at high speed. In addition, combining photonic and electronic stimuli in synaptic devices allows for the integration of synaptic functions with biometric sensing elements, including vision, auditory, and olfactory sensors [15, 16, 17]. In addition, the light‐sensitive photosensors function in a way that is comparable to the photoreceptors found in the biological retina, as both are capable of converting light signals into electrical signals [18]. This type of device, capable of replicating the functions of biological eyes and operating independently, may facilitate the development of future machine vision systems [19]. Park et al. [20] have expanded the human visual spectrum to include ultraviolet (UV) light, and this advancement holds potential for healthcare applications by integrating UV selectivity with dose‐calculation methodologies. Optical modulation, as an innovative gate‐control approach for achieving multi‐terminal neuromodulation, may reduce thermal loss and enable streamlined operation [21]. The incorporation of light‐stimulated synaptic devices has the potential to enhance both the functionalities and system complexities of contemporary neuromorphic systems [22]. Photonic neuromorphic systems leverage the inherent speed and parallelism of light to achieve lower energy consumption per operation. Recent advances in photonic synapses have demonstrated per‐event energy consumption as low as approximately 10^−^^1^ ^7^ J, which is significantly lower than that of biological synapses (≈ 10 fJ) and conventional electronic devices [3, 23].

At the same time, emerging applications such as wearables, displays, and brain‐machine interfaces require devices that operate with low power consumption, as well as being transparent and flexible. Moreover, for photonic‐enabled neuromorphic devices, the transparent nature of the devices is preferable as photonic devices rely on precise control and propagation of light [25, 26, 27]. In this context, the term “transparent” describes devices that effectively allow visible light to pass through them with a high level of transmittance, usually exceeding 70%–80%. In evaluating the devices, it is essential that they achieve a visible transmittance of around 70%–80%. This level of transmittance is necessary for the devices to be recognized as functioning transparently, allowing light to pass through while maintaining a clear view. This range is significant because an 80% transmittance level is commonly observed in practical applications, such as in display windows, where maintaining high visibility while also providing functional electronic capabilities is essential [28, 29, 30]. This means that a significant amount of light can travel through the material, making it clear and allowing for visibility of objects on the other side. This review offers an in‐depth exploration of optically transparent devices that function effectively in the visible to near‐infrared (NIR) spectrum. It highlights the unique properties of these devices, which enable them to blend effortlessly with various technological systems. By analyzing the potential applications and benefits of these transparent devices, the review underscores their significance in enhancing visual clarity, improving light manipulation, and enabling innovative design solutions across a range of fields, including telecommunications, medical imaging, and consumer electronics. Incorporating opaque components can result in increased absorption, scattering, or reflection, which may compromise signal fidelity and reduce overall device efficiency. Transparency ensures that neuromorphic elements do not block or scatter light, allowing co‐integration with cameras, waveguides, displays, or imaging optics. Invisible circuits enable the development of fully transparent wearable devices, such as smart contact lenses and heads‐up displays, that do not obstruct the user's wide‐field‐of‐view [31, 32, 33, 34, 35, 36]. Transparent photonic synapses can be placed directly over photodetectors or light‐emitting elements, performing preprocessing (e.g., edge detection, gain control) in the optical domain before any digitization. Furthermore, sustaining elevated optical transmittance in transparent devices minimizes insertion losses, thereby decreasing the necessary optical power for synaptic operations and facilitating system‐wide energy efficiency improvements. The objective of neuromorphic photonic processors is not to supplant conventional computers; rather, it is to facilitate applications beyond the capacity of traditional computing technology, particularly those demanding low latency, high bandwidth, and minimal energy consumption [37, 38]. Ultrafast neural networks can be applied in the following areas:

- Facilitating significant advancements in Physics: qubit readout classification [39], classification of high‐energy particle collisions [40], and plasma control within fusion reactors [41].
- Nonlinear programming: addressing nonlinear optimization challenges in fields such as robotics, autonomous vehicles, and predictive control [42], as well as solving partial differential equations [43].
- Acceleration of machine learning processes: including vector‐matrix multiplication [44], deep learning inference [45], and ultrafast or online learning methodologies [46].
- Advanced signal processing: including wideband radio frequency (RF) signal analysis [47], and fiber‐optic communications [48].

Photonic artificial NNs introduce novel demands for optoelectronic devices and photonic materials, necessitating the capability to store substantial data volumes while integrating synaptic functionalities to enable in‐memory computing. In recent developments, two‐dimensional (2D) materials have gained prominence as candidates for neuromorphic device applications, offering a practical approach to constructing ultrathin memristive synapses [49, 50, 51, 52, 53, 54]. 2D materials are generally defined as crystalline substances comprising a single atomic layer. Examples include the semimetallic graphene, the insulating hexagonal boron nitride (*h*‐BN), semiconducting transition metal dichalcogenides (TMDs), black phosphorus (BP), and group IV monochalcogenides such as SnS_2_, MoS_2_, SnSe, GeSe, and GeS [55]. The atomic‐scale thickness of 2D materials enables device miniaturisation, facilitates the implementation of high‐density crossbar arrays, and supports the development of synergistic heterostructured devices via van der Waals coupling [56, 57, 58]. At present, the majority of reported neuromorphic devices exhibiting synaptic functionalities are stimulated by electrical signals. However, recent research indicates that 2D materials and their hybrid heterostructures represent an optimal platform for non‐volatile photonic memory, due to their robust light‐matter interactions and pronounced photogenerated charge trapping resulting from their exceptionally high surface‐to‐volume ratio [59, 60, 61, 62, 63]. Furthermore, the wide range of available material types enables coverage across the electromagnetic spectrum from ultraviolet to infrared. As a result, 2D materials‐based photonic neuromorphic devices offer diverse applications, including image sensors for artificial vision [16], light‐gated memristors for logic operations [64], and photonic neural networks for neuromorphic systems [17].

Owing to the considerable promise of emerging 2D materials for optoelectronic applications, extensive research efforts have focused on demonstrating and advancing their integration into photonic memory and photonic synapse devices. In 2D materials‐based neuromorphic and synaptic devices, both light signals and gate voltage are commonly applied for programming or erasing operations, while the electric source–drain voltage is used to measure the read‐out current. The synaptic weight is represented by the conductance of the 2D material's channel. To date, most of the research on two‐terminal photonic memristors and three‐terminal floating gate photonic memory has primarily focused on materials such as molybdenum disulfide (MoS_2_), tungsten diselenide (WSe_2_), and black phosphorus (BP). The two‐terminal vertical memristor, leveraging the Schottky barrier modulation and conductive filament formation, offers the advantages of lower switching voltages and higher integration density. The three‐terminal lateral photonic memristor typically exploits the photo‐generated charge trapping/detrapping on the floating gate, which provides more design flexibility and new functionality. Beyond this device‐level emulation of synaptic dynamics, these 2D materials photonic synaptic devices have also been used to build photonic artificial NNs. Subsequently, pattern recognition tasks have been verified by these artificial NNs, where image pre‐processing and color‐mixed pattern classification are applied [16, 17] In addition to 2D materials, transparent oxide and doped‐oxide compounds such as In_2_O_3_, ZnO, TiO_2_, Sn‐doped In_2_O_3_, Al‐doped ZnO, and In‐Ga co‐doped ZnO provide a highly adaptable and complementary metal‐oxide semiconductor (CMOS)‐compatible foundation for the development of neuromorphic photonic devices. These materials distinctively combine optical transparency with both synaptic and memory functionalities. Transparent oxide materials possess wide band gaps (>3 eV), which provide high visible transmittance levels (>85%–90%). Modulation of carrier density, whether electrical or optical properties, can adjust the refractive index and absorption characteristics through the epsilon‐near‐zero (ENZ) effect, resulting in pronounced, low‐energy optical nonlinearities. Such properties make them well‐suited for energy‐efficient, transparent neuromorphic devices. Materials including HfO_2_, TiO_2_, and SnO_2_ demonstrate either filamentary or interface‐type resistive switching. When these films are incorporated within a transparent waveguide or positioned above or below the waveguide core, they enable the storage of synaptic weights through modulations in refractive index or optical loss. Utilizing transparent oxide materials exclusively enables the development of fully transparent, energy‐efficient neuromorphic photonic devices. These devices, which can range from single synaptic elements to integrated neural networks, are particularly well‐suited for applications in edge artificial intelligence, wearable electronics, and implantable brain‐machine interfaces.

Transparent neuromorphic devices require active materials that not only facilitate the conduction and modulation of electrical and optical signals but also allow efficient light transmission. In this context, we focus on 2D materials and transparent oxides, both of which offer high optical transmittance alongside advanced electronic capabilities. In this review, we discuss recent advances in 2D and oxide photonic materials for transparent, energy‐efficient neuromorphic devices, including neural networks, artificial synapses, and memory devices. We highlight key performance metrics (speed, energy) and emerging applications in edge computing and bio‐interfacing with the strategy of transparent neuromorphic device fabrications for future energy‐efficient neuromorphic devices.

## 2 Fundamentals of Photonic‐Based Neuromorphic Computing: Fundamentals and Limitations

### 2.1 Fundamentals of Conventional Computing and Its Limitations

As electronic devices continue to evolve and computing performance improves, there is an ongoing demand for smaller devices to support this growth and maintain the upward trend over time [65]. Currently, most computers are built using the traditional von Neumann architecture. This architecture, also known as the Princeton Architecture, is the foundational organizational structure of digital computers, based on the principles proposed by the mathematician John von Neumann. It treats instructions, which are computer programs, as a special type of data, storing both instructions and data at different addresses in the same memory. The von Neumann architecture is characterized by its use of a binary system and the execution of computations in a procedural order. The creation of this architectural style established the basis for modern computer architecture concepts [66]. Generally, a von Neumann architecture‐based computer is mainly composed of a memory bank for storing data and instructions and a CPU for performing nonlinear operations and connecting transmission between the two [67, 68, 69]. The limitations of hardware fabrication technology and inherent structural issues, such as the “memory wall,” hinder this architecture. As a result, the conventional von Neumann architecture is not well‐suited for processing large amounts of data in the era of AI [70]. It is crucial to propose alternative architectures that can scale beyond von Neumann to overcome computing bottlenecks.

Due to the continuous growth in data and computations, traditional computing struggles to keep pace with current developments. The impact of reducing device size on traditional computational performance has diminished over time, and Moore's Law, which has driven the advancement of computational capability in electronic devices, is inevitably reaching its conclusion [71]. In this context, computers based on the von Neumann architecture encounter issues such as the “memory wall,” which restricts system performance. This limitation complicates the ability to meet the modern demands for efficiency, energy consumption, density, and cost in high‐performance computing [72]. During computing, data is frequently transferred between the memory unit and the processor unit. This process often leads to noticeable delays, which can significantly impact overall system performance. Additionally, these data transfers consume a considerable amount of energy, contributing to the overall power consumption of the device. Efficient management of this data flow is crucial for optimizing speed and minimizing energy usage in modern computing systems. Due to the von Neumann architecture, battery‐constrained edge devices, such as always‐on sensors, wearables, and implantable brain‐machine interfaces (BMIs), have strict system‐level energy and heat budgets in the µW to mW range. Conventional digital stacks often need extensive quantization, pruning, or cloud offloading to satisfy these constraints, each with associated costs in latency, privacy, or accuracy. This encourages the development of alternative architectures that integrate memory and computation (in‐memory/in‐sensor computing) while utilizing parallel physical channels (e.g., optical wavelength‐division multiplexing (WDM)/spatial multiplexing) to decrease wall‐clock latency and the energy required per effective neural operation [73].

Given the end of Moore's Law and the limitations of the von Neumann architecture, it is crucial to address the core architectural bottleneck in modern computing. There is a pressing need to explore alternative architectures and paradigms to develop non‐von Neumann systems, which can significantly enhance computing performance.

### 2.2 Fundamentals of Neuromorphic Computing

**FIGURE 2:**
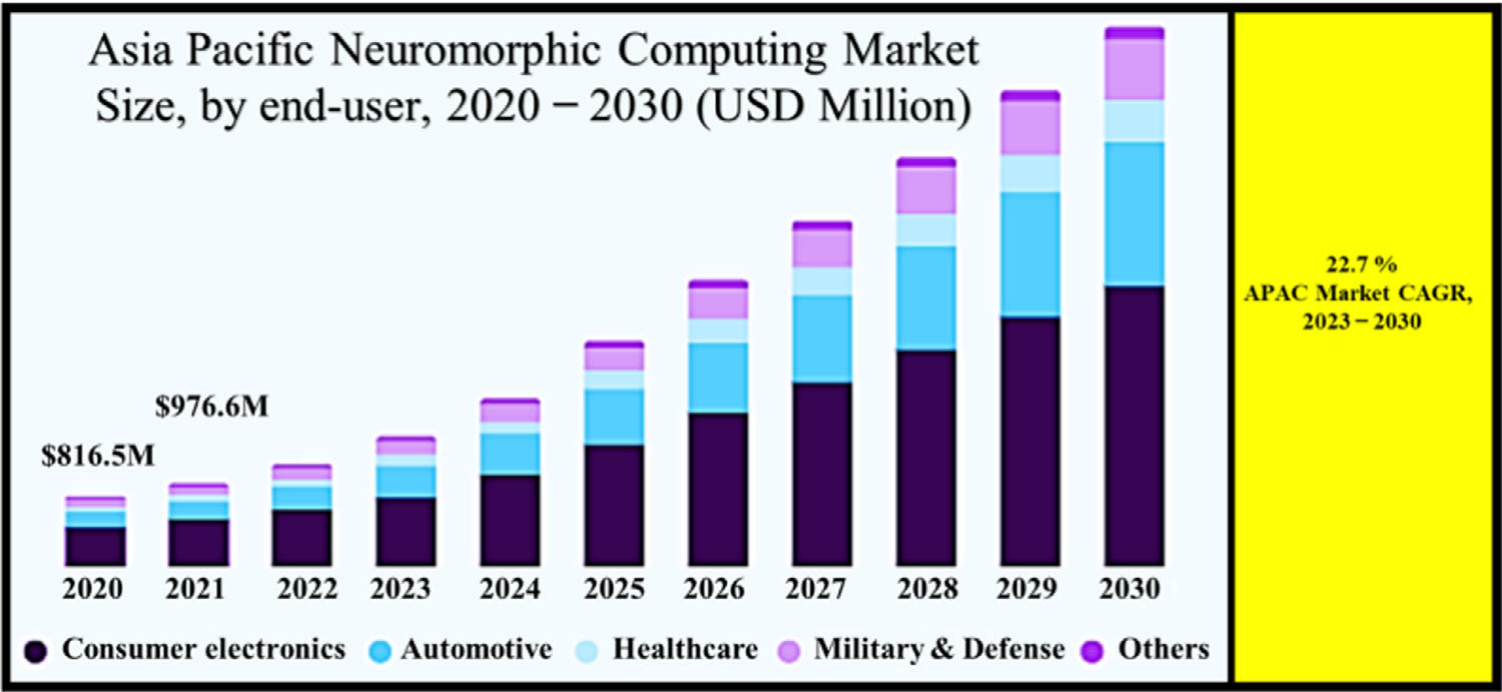
Neuromorphic computing market. Reproduced with permission [84]. Copyright 2023, Multidisciplinary Digital Publishing Institute.

Current computer technology is encountering two significant challenges: the memory wall effect of the “von Neumann” architecture results in low energy efficiency [74, 75], and Moore's Law, which has historically driven semiconductor development, is anticipated to come to an end in the next few years [76, 77]. On the other hand, traditional architecture converts the processing of high‐dimensional information into one‐dimensional processing focused solely on time, resulting in low efficiency and high energy consumption [78]. The global neuromorphic computing market reached a significant valuation of USD 4,237.7 million in 2022, marking a notable growth trajectory. Projections indicate that this market is poised for a significant expansion, with a projected compound annual growth rate (CAGR) of 21.2% from 2023 to 2030 [79], as illustrated in Figure 2. In 1989, Carver Mead from Caltech proposed the concept of “neuromorphic engineering,” also known as brain‐like computing, in his book titled “Analog VLSI Implementation of Neural Systems,” which employs sub‐threshold analog circuits to simulate Spiking Neural Networks (SNN) [80]. It is widely recognized that the nervous system of mammals, particularly in humans, is among the most efficient and resilient structures found in nature. The human brain consists of a vast number of connections and demonstrates strong parallel processing abilities. It contains approximately 100 billion neurons and 10^15^ synapses, yet it only consumes about 20 W of energy [81]. Neurons establish biological connections within a few milliseconds and possess excellent fault‐tolerance mechanisms for individual component failures [82]. For computer scientists, there are tremendous similarities between neural systems and digital systems. Components, including the cell body, dendrites, axons, nerve terminals, and synapses, make up a neuron unit. The core component of a neuron is its cell body, which contains a nucleus and has a radius ranging from 2 to 60 microns. On the surface of the cell body, there are two types of cell processes of varying lengths: a single long axon and multiple short dendrites. Excitatory transmission between neurons occurs through the axons and nerve terminals, ultimately reaching the synapse, which is the connection point between neurons [83]. Neurons serve various functions and together form a complete nervous system that can effectively receive, integrate, and transmit information. This process is fundamental to how the nervous system learns and adapts. While brain neural networks possess different capabilities for information processing and logical analysis at various levels, they operate as a coordinated and unified system, with each part closely interconnected [83].

The neuromorphic computer is an innovative model that mimics the brain's neural network using ultra‐large‐scale pulses and enables real‐time communication [85]. Neuromorphic computers aim to replicate the high performance, low power consumption, and real‐time processing capabilities of biological brain neural networks. They utilize large‐scale CPU/GPU clusters to implement these neural networks. In a CPU cluster, each thread is assigned to simulate a corresponding neuron, allowing thousands of threads (neurons) to operate in an orderly and coordinated manner, effectively forming a complete large‐scale neural network.

The implementation of neuromorphic computing at the hardware level can be achieved using oxide‐based CMOS devices like memristors, spintronic memories, threshold switches, transistors, and others. In 2008, Hewlett‐Packard (HP) developed a memristor prototype that could mimic the function of synapses and demonstrated the first hybrid circuit integrating memristors and silicon materials [86]. Memristors enable the creation of highly energy‐efficient, durable, and self‐learning processors. This unique capability has garnered significant interest in fields such as neural networks and artificial intelligence. Numerous research groups are currently investigating the application of memristors to develop more efficient neural network architectures.

### 2.3 Challenges and Limitations Encountered by Existing Neuromorphic Computing Devices

**FIGURE 3:**
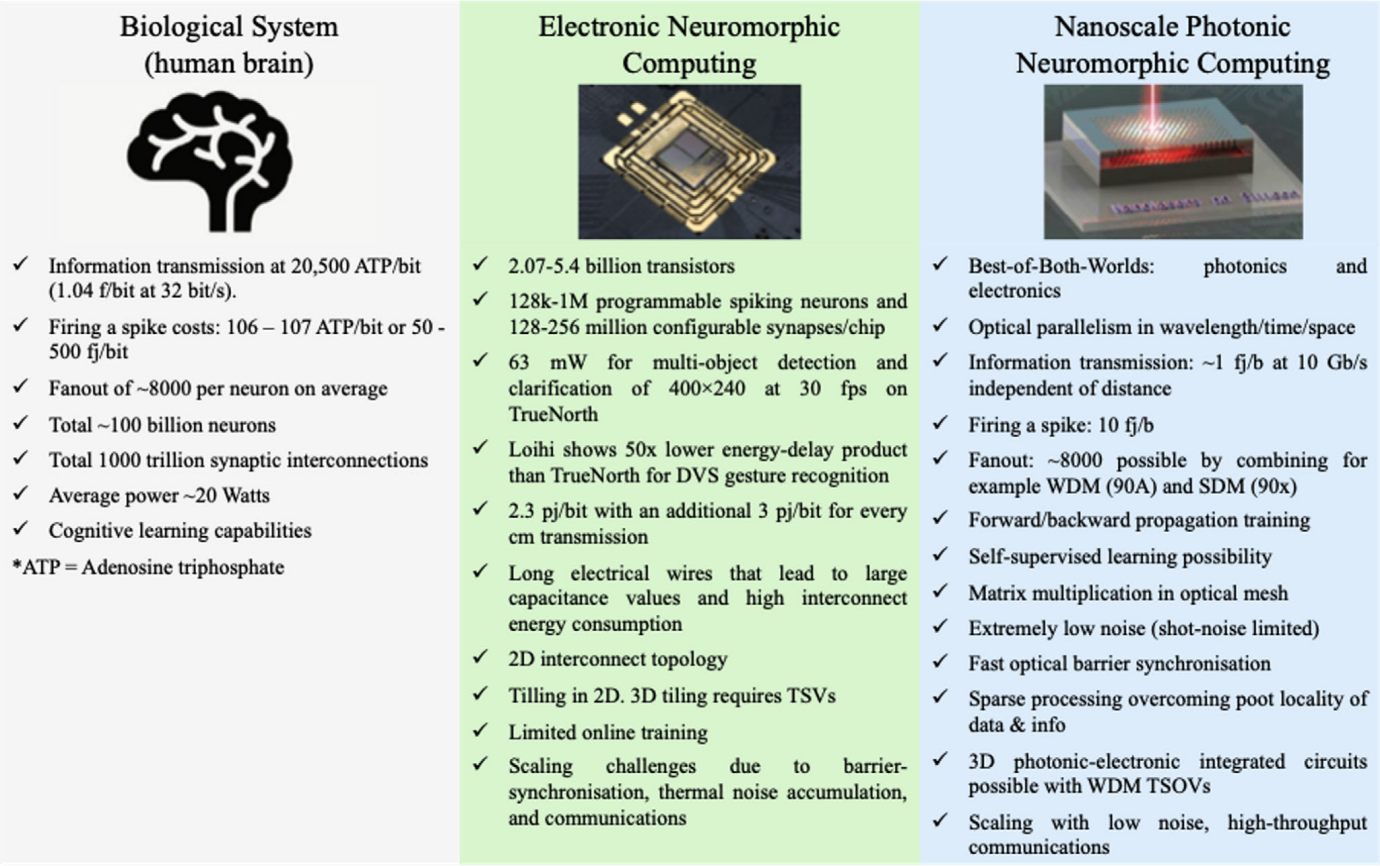
Comparisons of a biological cognitive system (the human cortex and brain), CMOS‐based electronic neuromorphic computing, and the latest photonic neuromorphic computing. Reproduced with permission [89]. Copyright 2022, American Institute of Physics. Reproduced with permission [24]. Copyright 2025, Wiley‐VCH.

The limitations of traditional brain‐inspired computing primarily include three areas: hardware limitations, software and algorithm limitations, and practical application limitations. They will be discussed in detail below in the text and in Figure 3, which compares biological brains with electronic and photonic neuromorphic computing systems. Despite the claims of neuromorphic computing regarding low power consumption, current hardware generally consumes more energy than biological nervous systems. Years of research and significant funding have gone into various forms of pattern recognition. Despite notable advancements, synthetic electronic systems still do not match the capabilities of human perception in specific areas [87]. Materials significantly influence energy consumption in neuromorphic computing systems. The inherent properties of conventional conductors, such as their conductivity and resistivity, contribute to energy inefficiencies. When electrical currents are transmitted through these materials, a notable amount of energy is lost as heat due to resistive effects. This energy dissipation not only affects the overall performance of neuromorphic devices but also imposes limitations on their scalability and efficiency. Therefore, selecting advanced materials with improved conductivity and reduced resistivity is essential for enhancing energy efficiency in neuromorphic computing applications. Furthermore, the size factor significantly constrains the energy consumption potential of traditional conductive materials. As these materials increase in dimension, their ability to efficiently conduct energy diminishes, leading to heightened energy losses and reduced overall performance. This limitation poses a challenge for the development of advanced applications that require optimal energy efficiency. As neuromorphic computing devices scale down to increasingly compact sizes, the influence of quantum and thermal effects is likely to become more significant. These phenomena could have a considerable impact on energy efficiency, potentially altering how these advanced technologies operate and perform in real‐world applications [88]. Conventional electronic systems, as shown in Figure 3, consume energy levels that are three orders of magnitude higher than those of biological and photonic systems.

In the context of computing systems and networks, “communication overhead” refers to the additional resources and time required for the transmission of information beyond the actual data being sent [90]. This overhead can include various factors such as the energy consumed by networking protocols, the processing time needed to manage data packets, and the bandwidth utilized for control messages. Understanding communication overhead is essential for optimizing network performance, as it impacts the efficiency of data transfer and overall system responsiveness. By minimizing communication overhead, systems can achieve faster data exchange and improve user experience in applications ranging from cloud computing to real‐time communications. In the realm of neuromorphic computing, several important factors come into play. A primary concern is data transmission, which plays a crucial role in the simulation of neural networks. In these systems, information must travel through structures analogous to biological synapses, facilitating communication between simulated neurons. This process is inherently complex and often leads to increased energy consumption and latency during hardware implementation. Efficient data transfer is essential, as it directly impacts the overall performance and effectiveness of neuromorphic systems. Innovations in this area can help reduce these energy costs and improve the speed of processing, ultimately enhancing the capabilities of neuromorphic computing to mimic the efficiency of the human brain [91]. Additionally, neurons generally require specialized encoding and decoding techniques to accurately represent their activity. This necessity increases computational demands and energy consumption, impacting overall system efficiency. Furthermore, some neuromorphic computing models hinge on the precise timing synchronization between neurons to function effectively. This requirement not only complicates the design of these systems but also contributes to a significant increase in both communication and computational overhead, ultimately affecting the performance and scalability of neuromorphic applications [92]. In large‐scale neural networks, the transmission of information involves navigating intricate pathways that connect multiple neurons across various network layers. This complexity often requires the integration of specialized hardware and sophisticated algorithms to effectively manage the flow of data. To maintain the integrity and accuracy of the information being transmitted, it becomes crucial to implement robust error detection and correction mechanisms. While these systems are vital for ensuring reliable communication, they also contribute to increased communication overhead. This overhead can manifest as additional processing time and resource usage, highlighting the trade‐offs involved in achieving precise information transfer within these expansive neural architectures [93].

Parallelism and synchronization play crucial roles in determining the power consumption of neuromorphic computing systems. In contrast to traditional electronic architectures, which encounter notable limitations in both areas, neuromorphic designs aim to address these challenges. One of the primary constraints in traditional electronics is related to parallelism. Electronic devices are frequently restricted by the bandwidth of their circuits, which hinders their ability to execute highly parallel computations effectively. This bandwidth limitation can lead to bottlenecks, where the flow of data through the circuit becomes a constraining factor in processing speed and efficiency. As a result, achieving optimal performance in tasks that require simultaneous operations across multiple processors can be difficult. In neuromorphic computing, the goal is to mimic the efficiency of biological neural networks, which naturally excel in parallel processing. By leveraging specialized architectures that facilitate greater parallelism and flexibility in synchronization, neuromorphic systems can potentially reduce power consumption while enhancing processing capabilities. Understanding and overcoming the limitations faced by traditional electronics is crucial for developing more efficient and powerful neuromorphic devices. Additionally, in highly parallel electronic systems, electromagnetic interference can become a serious problem, limiting overall performance [94]. Secondly, achieving global clock synchronization in large‐scale electronic systems presents significant challenges due to its complexity and the high energy demands involved. Ensuring that all components operate in perfect harmony requires intricate coordination and precise timing, which can become increasingly difficult as the scale of the system expands. This process not only consumes substantial energy resources but also necessitates sophisticated technologies to maintain accurate synchronization across diverse and distributed elements [95]. Additionally, the transmission speed of electronic signals in wires is inherently restricted, leading to challenges in achieving precise synchronization within systems. These limitations collectively impede the effectiveness of traditional electronic devices, particularly concerning their capacity for parallel processing and timing accuracy. In stark contrast, photonic systems harness the unique properties of light to attain a remarkable level of parallelism. By employing techniques such as inherent fanout and WDM, photonic systems can simultaneously transmit multiple signals across different wavelengths, as illustrated in Figure 3. This capability not only enhances data throughput but also optimizes synchronization, making photonic technology a superior alternative in the realm of advanced communication systems.

### 2.4 Fundamentals of Photonics

Photonics is a dynamic and multidisciplinary field that encompasses the comprehensive study of light, including its propagation and the intricate ways it interacts with various materials. This area of research encompasses various forms of electromagnetic radiation, with a primary focus on visible light, UV, and IR wavelengths. Photonics plays a crucial role in several cutting‐edge applications, such as fiber optic communications, where it enables high‐speed data transmission over long distances, and medical imaging technologies, which enhance diagnostic capabilities and treatment precision. Additionally, advancements in laser technology, including applications in manufacturing, materials processing, and even entertainment, highlight the transformative potential of photonics. By investigating and leveraging the properties of light, scientists and engineers in this field strive to unlock innovative solutions that drive progress across diverse sectors, from telecommunications to healthcare and beyond. This section provides a concise yet rigorous account of the photonics fundamentals that are directly relevant when designing photonic neuromorphic hardware. This work establishes a connection between fundamental principles of optics and photonics and the specific metrics used at both the device and system levels in neuromorphic computing. The field of photonics encompasses a diverse array of scientific concepts and technological advancements, making it vital for a wide range of applications, from telecommunications to advanced computational systems. By understanding how these optical principles can be applied to neuromorphic computing, we can better harness the power of light‐based technologies to create more efficient and effective computing systems that mimic the neural processes of the human brain. The key focus areas in this field are:

i. Optical Sensing: This technology leverages light to detect and measure various physical and chemical properties, such as temperature, pressure, and chemical composition. By utilizing advanced sensors and photonic devices, optical sensing offers high sensitivity and precision, making it applicable in diverse areas like environmental monitoring, medical diagnostics, and industrial process control. ii. Optical Interconnection: This area involves the use of light to facilitate data transfer within and between electronic devices. Optical interconnections significantly improve data transfer rates compared to traditional electrical interconnections, enabling faster and more efficient communication in computing systems. This technology supports the growing demand for high‐speed data processing and is essential for applications such as data centers, high‐performance computing, and telecommunications networks. iii. Optical Communication: This critical field focuses on using light signals to transmit information over long distances. Optical communication systems, such as fiber optic networks, provide high bandwidth and low signal attenuation, making them ideal for long‐haul transmission. By employing techniques like wavelength division multiplexing, multiple data streams can be sent simultaneously over a single optical fiber, vastly increasing capacity and efficiency in communication networks vital for the internet and telecommunication infrastructure.

By utilizing the unique properties of light, photonics plays a critical role in advancing technologies in telecommunications, healthcare, and manufacturing [96, 97]. Photonics is a vital technology in the development of optical computing systems, which leverage light rather than electricity to process and transmit information. Unlike conventional electronic computers that rely on the flow of electric current, optical computing harnesses the properties of light to perform calculations at incredible speeds. This method not only enhances processing speed but also significantly reduces energy consumption, making it a more efficient alternative. The use of photons for data transmission enables parallel processing capabilities, allowing for the simultaneous handling of multiple data streams. This characteristic is particularly advantageous for applications requiring high bandwidth, such as data centers and telecommunications. Additionally, optical computing has the potential to minimize heat generation, thus improving overall system performance and longevity. As research and development in photonics continue to advance, the integration of optical computing systems could herald a new era in information technology, positioning it as a transformative force in various fields. Optical computing utilizes the unique properties of light to perform intricate calculations at astonishing speeds, significantly outperforming traditional electronic computing methods. By employing photons instead of electrons, optical systems can process vast amounts of data simultaneously, leading to faster computation times. Additionally, these systems consume considerably less power, making them a sustainable option for modern applications. This efficiency is particularly advantageous in environments such as data centers, where rapid data handling is crucial, and energy costs can be substantial. As the demand for high‐performance computing continues to grow, the potential of optical computing in revolutionizing data processing and reducing energy consumption becomes increasingly significant. It is important to understand that electrons and photons are two fundamental particles that play crucial roles in the physical universe. Electrons, which carry a negative electric charge, are key components of atoms, influencing chemical bonds and the behavior of matter. Photons, on the other hand, are massless particles of light, responsible for electromagnetic radiation and conveying energy across distances. Together, these elementary particles form the foundation of our understanding of the natural world and the interactions that govern it. Electrons are a type of particle known as fermions, which are distinguished by their half‐integer spins (such as 1/2, 3/2, and 5/2). One of the key principles that governs fermions is Pauli's exclusion principle, which states that no two fermions can occupy the same quantum state simultaneously. This principle is fundamental to the structure of matter, as it explains a variety of phenomena, including the arrangement of electrons in atoms and the stability of atomic nuclei. In addition to electrons, other examples of fermions include protons and neutrons (which make up atomic nuclei), neutrinos (subatomic particles that are very light and interact weakly with matter), and quarks (the fundamental constituents of protons and neutrons). The behavior of these particles is essential to understanding the physical universe, particularly in fields like particle physics and cosmology. Fermions are distinct from bosons, another class of particles that have integer spins (such as 0, 1, 2, etc.). Unlike fermions, bosons do not follow Pauli's exclusion principle, which allows multiple bosons to occupy the same quantum state simultaneously. This property leads to phenomena such as superconductivity and Bose‐Einstein condensates. The wave function of electrons is prescribed by the Schrodinger equation (Equation (1), while that of photons is derived from Maxwell's equations (Equations 3–6). Electrons possess a defined mass and exhibit a drift velocity that is constrained by their interactions with surrounding materials, such as conductors. In contrast, photons are fundamentally massless particles that move unimpeded at the speed of light, enabling them to traverse vast distances across the universe almost instantaneously. The Schrödinger equation for the electron is:

(1) $$E\psi =-\frac{\hbar^{2}}{2\mu }\nabla^{2}\psi -\frac{q^{2}}{4\pi \varepsilon_{0}r}\psi$$

where *E* is energy, *q* is the electron charge, ***r*** is the position of the electron relative to the nucleus, *r* = | ***r*** | is the magnitude of the relative position, the potential term is due to the Coulomb interaction, wherein *𝜖_0_* is the permittivity of free space, and

(2) $$\mu =\frac{m_{e}m_{p}}{m_{e}+m_{p}}\;$$

is the two‐body reduced mass of the hydrogen nucleus (just a proton) of mass *m_p_* and the electron of mass *m_e_*.

As researchers delve deeper into the potential of photonics, advancements in this field are poised to fundamentally transform information processing in the digital era. Photonics, which involves the use of light to transmit and manipulate data, offers remarkable advantages over traditional electronic systems. These advancements could lead to faster data transmission speeds, improved energy efficiency, and enhanced capabilities for handling large volumes of information. By harnessing the unique properties of light, such as its speed and bandwidth, scientists are exploring innovative applications across various sectors, including telecommunications, computing, and medical devices. The continued development of photonic technologies could significantly reshape how we interact with information, paving the way for a future where data processing is quicker, more efficient, and more sustainable [98, 99]. Maxwell's equations are fundamental in understanding light‐matter interactions. These four interconnected equations describe the behavior of electric and magnetic fields and predict the self‐propagating nature of electromagnetic waves, with light serving as one of the most significant examples. In a vacuum, light travels in waves and can exhibit various properties. When light encounters matter, these interactions become more complex. Maxwell's equations delineate the processes of reflection, where light bounces off surfaces; refraction, where light bends as it passes through different media; absorption, where light energy is taken in by materials; and transmission, where light passes through substances. Each of these phenomena is crucial for applications ranging from optical devices to understanding fundamental physical principles in both classical and quantum physics. By analyzing these interactions using Maxwell's equations, scientists can gain deeper insights into the nature of light and its behavior in various environments. The boundary conditions established by Maxwell's equations at the interfaces between different media play a crucial role in shaping the distributions of electromagnetic fields. These conditions dictate how electric and magnetic fields interact and transition across various materials, leading to specific behaviors in the optical responses of those materials. Understanding these interactions is essential for predicting how light will behave when it encounters different surfaces, which is fundamental in fields such as optics, telecommunications, and materials science. By analyzing these electromagnetic field distributions, we can gain deeper insights into phenomena such as reflection, refraction, and the absorption of light, ultimately influencing the design and application of various optical devices. Maxwell's equations play a fundamental role in understanding and quantifying the dielectric and magnetic properties of materials. These properties are characterized by two key parameters: permittivity, which measures a material's ability to store electrical energy in an electric field, and permeability, which gauges its ability to conduct magnetic field lines. Together, these equations provide a comprehensive framework for analyzing how materials respond to electric and magnetic fields, making them crucial in fields such as electromagnetics, material science, and electrical engineering. These properties significantly influence the phase velocity of electromagnetic waves as they travel through different media, leading to important phenomena such as dispersion, where different wavelengths of light separate, and polarization, which refers to the orientation of light waves. The underlying equations not only describe the conservation of energy and momentum within electromagnetic fields but also provide a comprehensive framework for understanding how light interacts with material particles. This interaction results in forces and torques that light can exert on these particles, a principle that is foundational for advanced technologies like optical trapping and manipulation. These technologies utilize focused light to control and manipulate small particles, enabling applications in fields such as biophysics, materials science, and nanotechnology. Equations (3)–(6) are Maxwell's equations that dictate the behavior of electromagnetic waves:

(3) $$\nabla .D\;=4\pi \rho_{f}$$

(4) ∇.B=0

(5) $$\nabla \times E\;=-\frac{\partial B}{\partial t}$$

(6) $$\nabla \times H\;=J_{f}+\frac{\partial D}{\partial t}$$

where ***D*** is electric flux density, ***B*** is magnetic flux density, ***E*** is electric field intensity, and ***H*** is magnetic field intensity. Maxwell's equations serve as the cornerstone of classical photonics, governing the behavior of electromagnetic fields and their interactions with matter. These equations describe how light propagates, reflects, refracts, and interacts with different materials, forming the theoretical basis for various technologies in optical communication and imaging. In the development of efficient optical neural networks (ONNs), which leverage the principles of light to perform complex computations, the integration of nanoscale components is crucial. These tiny devices, often on the order of nanometers, enable precise manipulation and processing of light signals with high speed and minimal energy loss. By utilizing advanced materials and innovative design techniques, researchers aim to optimize these components for more effective signal transmission and processing in ONNs, ultimately leading to breakthroughs in artificial intelligence and machine learning applications. Diffraction and interference of light at the nanoscale are fundamental phenomena that significantly enhance the functionality of optical components. These effects enable precise manipulation of light signals, which is essential for the encoding, processing, and transmission of information within modern optical networks. When light waves encounter obstacles or slits that are comparable in size to their wavelength, diffraction occurs, resulting in a spreading of the waves and the formation of characteristic patterns. Similarly, interference arises when two or more light waves overlap, leading to the emergence of regions of constructive interference, where the waves reinforce each other, and regions of destructive interference, where they cancel one another out. Both diffraction and interference are based on the superposition principle. This principle asserts that when multiple waves overlap, the resultant wave can be described as the sum of the individual wave amplitudes at each point in space. Understanding these effects at the nanoscale is crucial for the development of advanced technologies such as photonic devices, sensors, and communication systems, where precise control over light is vital for efficient operation and increased performance.

### 2.5 Inclusion of Photonic Components in Neuromorphic Computing Devices

In semiconductor physics, the interplay between electrons and photons is fundamental to the functioning of various devices. A diode laser and a light‐emitting diode (LED) are both examples of semiconductor devices that produce light when an electrical current flows through them. These devices rely on the properties of semiconductors, which can conduct electricity under certain conditions. When an electric field is applied, electrons within the semiconductor material gain energy and move into the conduction band. At the same time, they leave behind voids known as electron holes. This phenomenon creates a situation where electrons can recombine with these holes. As they do so, the excess energy released during this recombination process manifests as photons or light. The significant breakthrough that supports this understanding was made by Albert Einstein in 1916, when he predicted that this recombination process would lead to the emission of light. This principle is at the core of how both diode lasers and LEDs operate, making them essential components in various applications, from lighting to communication technologies. Neuromorphic computing aims to imitate brain‐like information processing through networks of artificial neurons and synapses. Integrating photonic components into neuromorphic hardware can help overcome significant limitations of electronic systems by utilizing the ultrafast, parallel capabilities of light. Optical interconnects provide extremely high bandwidth and minimal latency, allowing neural systems to function at speeds far exceeding those of conventional electronics [100]. Silicon photonic networks have shown processing rates that are 6 to 8 orders of magnitude higher than those of electronic networks [101, 102]. Importantly, silicon photonics effectively combines established CMOS fabrication techniques with the ultra‐high‐speed and low‐loss benefits of light, paving the way for large‐scale integrated neuromorphic chips. In contrast to opaque, resistance‐limited electronic chips, photonic components can even be transparent or flexible, opening new form factors for neuromorphic systems [103, 104].

*Communication (Interconnect):* Electronic neuromorphic chips use metal wires whose bandwidth is limited by capacitance and crosstalk. By contrast, optical waveguides and fibers provide extremely high bandwidth and negligible crosstalk, enabling parallel signal routing with ultra‐low latency.

*Operating Speed:* Photonic components (e.g. modulators, lasers) can switch and transmit at GHz‐to‐THz rates. For instance, vertical cavity surface‐emitting laser (VCSEL) neurons emit sub‐nanosecond spikes for ultrafast image processing, whereas electronic transistors are typically limited to tens of GHz.

*Energy Efficiency:* Optical synapses rely on photo‐induced conductance changes rather than resistive heating, which can reduce energy per operation. In practice, photonic synapses have been shown to exhibit plasticity under low‐energy light pulses [103].

*Integration:* Silicon photonic platforms integrate high‐quality optical components (rings, Mach‐Zehnder interferometers (MZIs), modulators) with CMOS electronics. This hybrid approach yields high‐density photonic neural circuits (e.g. interferometer arrays) while retaining the manufacturing benefits of electronics.

*Device Transparency*: Many photonic materials (e.g. transparent conducting oxides, 2D materials) enable fully transparent devices. All‐oxide photonic synapses on glass and 2D‐material photonic synapses with graphene electrodes have been demonstrated, unlike opaque silicon chips [104].

*Synaptic Plasticity:* Electronic synapses typically use voltage pulses to emulate plasticity. Photonic synapses can instead use light to directly induce plastic changes; for example, oxide synapses exhibit short‐ and long‐term potentiation (STP and LTP) under UV illumination, and 2D‐material synapses show persistent photoconductivity akin to biological learning.

These trends highlight why photonic components are attractive for neuromorphic devices. In the laboratory, a variety of photonic synapse and neuron implementations have been explored. For instance, Kumar et al. demonstrated a transparent, all‐oxide photonic synapse (In_2_O_3_/ZnO on FTO/glass) that shows memristive behavior and UV‐induced STP/LTP as well as paired‐pulse facilitation [103]. The synaptic conductance of this device can be optically potentiated and electrically depressed, directly mimicking key learning rules. Similarly, an all‐oxide NiO/TiO_2_ heterostructure (≈ 54% transparent) was shown to mimic basic visual‐cortex functions (orientation selectivity and spatiotemporal processing) under self‐biased optical excitation [105]. In a recent advance, Dong et al. reported a transparent planar photonic synapse based on 2D carbon heterostructures and graphene electrodes, enabling dual‐sided (≈ 360°) photosensing and synaptic plasticity [104]. This device achieved broadband neuromorphic vision (365–970 nm) and true synaptic behavior (STP, LTP, Paired‐pulsed facilitation or PPF) in a planar, transparent form. These and other experimental demonstrations show that photonic synapses can be made transparent or semi‐transparent, which is useful for integrating neuromorphic sensing and display (e.g. retinal implants or augmented‐reality devices).

*Photonic Synapses:* Photoresponsive materials (oxides, organics, 2D heterostructures) form the basis of optical synapses. For example, oxide heterojunction devices (NiO/TiO_2_, In_2_O_3_/ZnO) show plasticity under light [103, 105]. Organic bulk‐heterojunction films and perovskites have also been proposed for flexible, transparent photosynapses. All these devices adjust their conductance when illuminated, emulating synaptic weight updates in response to optical spikes.

*Photonic Neurons:* Nonlinear optical elements act as artificial neurons. Semiconductor laser neurons (e.g., VCSELs with saturable absorbers) have been shown to fire ultrafast spikes on threshold. Such photonic leaky‐integrate‐and‐fire (LIF) neurons can perform tasks like edge detection in nanoseconds. Integrated optical resonators (microrings, photonic crystals) can also implement neuron‐like thresholding or relaxation oscillators. Notably, architectures using chalcogenide phase‐change rings have been proposed as spiking neurons, where optical pulses switch a PCM element to simulate membrane integration and firing.

*Photonic Memory:* Nonvolatile photonic memory is crucial for weights. Integrating phase‐change materials (e.g., Ge_2_Sb_2_Te_5_) on photonic waveguides enables all‐optical memory. Pioneering work (Ríos et al.) deposited GST on Si_3_N_4_ microrings and MZIs, achieving tunable, nonvolatile photonic storage [100]. Later, multi‐level photonic memories (up to 3–8 bits) and 16 × 16 PCM waveguide arrays were demonstrated [106]. These PCM‐based synapses and memories change their optical transmission in a controlled way, effectively implementing plastic weights in an integrated photonic network.

*Integrated Photonic Circuits:* Beyond individual devices, full neural networks have been built on‐chip using photonic components. For example, Shen et al. (2017) implemented a photonic neural network using a mesh of MZIs to perform vowel classification [107]. Tait et al. and others have proposed systems using on‐chip modulators and wavelength‐multiplexed lasers to realize large‐scale synaptic arrays [108]. These demonstrations exploit the ultra‐high speed and low‐loss propagation of light for in‐memory vector‐matrix multiplication at light speed, potentially using transparent waveguides and planar optics for scalability. For clarity, Table 1 summarizes key differences between electronic and photonic neuromorphic components and gives examples of photonic implementations.

**TABLE 1:** Comparison of electronic vs photonic neuromorphic components and examples of photonic device implementations.

Component/Aspect	Electronic implementation	Photonic implementation (examples)
Communication	Electrical wires and interconnects, limited by RC delay and crosstalk (GHz–low GHz bandwidth)	Optical waveguides/fibers: low‐loss, parallel links with ultrahigh bandwidth (hundreds of THz)
Synapse (plasticity)	Resistive memristors (metal‐oxide films, floating‐gate FETs) or CMOS analog circuits	Photonic synapses: transparent oxide memristor (In_2_O_3_/ZnO) driven by UV light; 2D‐material planar photosynapse (PeG/graphene) with 360° sensitivity; Phase‐change (GST) on Si waveguide for optical STDP
Neuron (activation)	CMOS spiking/integrate‐fire circuits (transistor networks)	Photonic neurons: VCSEL or FP lasers with saturable absorbers (spiking LIF behavior); optical nonlinear resonators (e.g. microrings with PCMs) for thresholding
Memory / Storage	SRAM/DRAM, Flash or nonvolatile memristor arrays	Photonic memory: GST phase‐change films on Si_3_N_4_ waveguides/microrings; integrated PCM arrays (e.g. 16 × 16 photonic memory cells)
Integration / Scaling	Ultra‐dense VLSI CMOS, but limited by interconnect	Silicon photonic PICs (CMOS‐compatible) combining modulators, detectors, lasers; limited by optical wavelength but enabling massive WDM.
Transparency / Form	Opaque silicon chips with metal layers	Transparent/flexible: e.g. oxide layers on glass, 2D‐material synapses with graphene electrodes; supports see‐through or wearable neuromorphic devices.

Overall, photonic neuromorphic devices exploit optical physics (photoexcitation, phase‐change, nonlinear optics) to implement neuron/synapse functions. These components can be designed to be transparent or flexible, integrating with sensors and displays. The combination of ultrahigh speed, parallelism, and new material functionalities in photonic synapses/neurons is expected to enable neuromorphic systems with far higher throughput and potentially lower power than purely electronic designs.

## 3 Concepts and Architectures in Transparent Photonic Neuromorphic Computing Devices

Neuromorphic photonic systems leverage light‐based circuits to effectively simulate the functioning of neural networks. These advanced systems capitalize on the properties of optical transparency, which allows for exceptionally high bandwidth and the ability to process multiple signals simultaneously through parallelism. Additionally, they minimize crosstalk, or unwanted interference between signals, making them highly efficient for complex computations. By integrating photonic design with neuromorphic principles, these systems are poised to revolutionize fields such as artificial intelligence, machine learning, and data processing, offering significant advantages over traditional electronic counterparts [109]. Essential photonic building blocks include: waveguides (e.g., silicon or silicon‐nitride) for low‐loss signal routing, MZIs for tunable splitting and interference, microring resonators (MRRs) for wavelength‐selective filtering and weighting, nonlinear devices (e.g., semiconductor lasers, saturable absorbers) that produce neuron‐like spiking dynamics, and phase‐change materials (PCMs) on waveguides or resonators for nonvolatile optical memory and synaptic weights [84, 109].

### 3.1 Materials for Photonic Memory and Synapses

**FIGURE 4:**
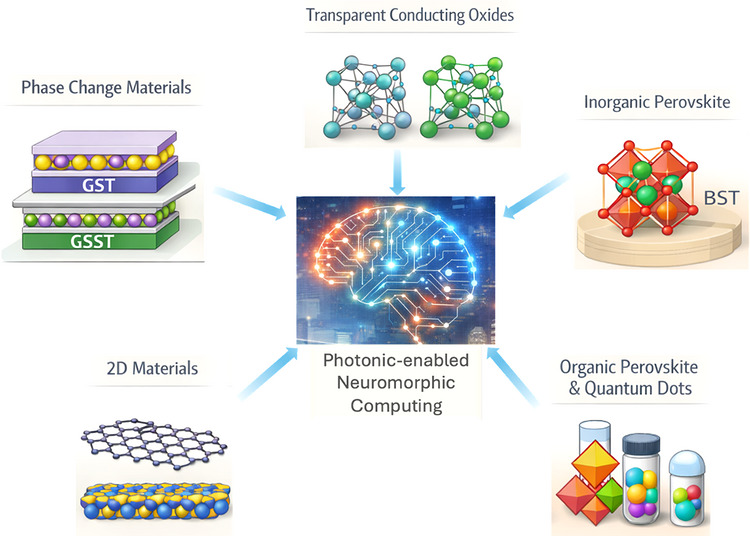
Comprehensive schematic of suitable materials for transparent photonic‐enabled neuromorphic computing devices. The figure illustrates five material categories: (i) Phase Change Materials (GST, GSST), (ii) Transparent Conducting Oxides, (iii) Inorganic Perovskite (BST), (iv) Organic Perovskite and Colloidal Quantum Dots, and (v) Two‐Dimensional Materials (Graphene, TMDCs).

Transparent neuromorphic photonic devices require specialized materials with wide optical band gaps. These band gaps are crucial for ensuring transparency in the visible and NIR spectral regions. Additionally, these materials must exhibit switchable electronic or photoelectronic responses, allowing for dynamic manipulation of their properties in response to external stimuli. This combination is essential for advancing the functionality and efficiency of these innovative devices. Figure 4 exhibits a comprehensive schematic of suitable materials for transparent photonic‐enabled neuromorphic computing devices. Researchers have been actively investigating a range of advanced materials, including wide‐bandgap PCMs, which are being studied for their capacity to change states and conduct electricity at high temperatures, which could revolutionize data storage and processing. Transparent oxides have also been explored for their ability to conduct electricity while allowing light to pass through, making them ideal for use in displays and solar cells. Additionally, 2D semiconductors are known for their unique electronic properties and potential applications in flexible electronics. Moreover, hybrid systems that combine the exciting properties of perovskites and quantum dots are being developed, offering promising avenues for next‐generation optoelectronic devices.

**FIGURE 5:**
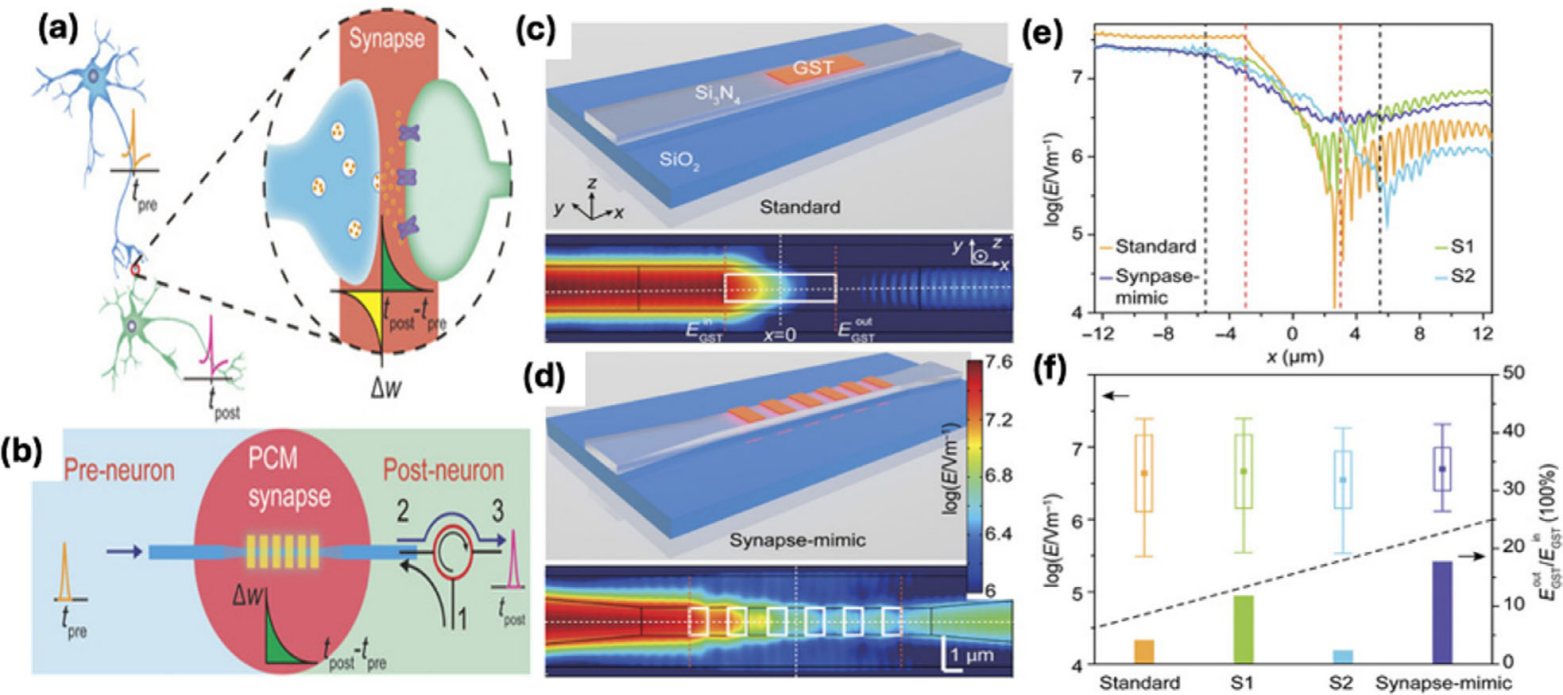
(a) Structure of neuron and synapse. Inset: Illustration of the synapse junction. (b) Schematic of the integrated photonic synapse resembling the function of the neural synapse. The synapse is based on a tapered waveguide (dark blue) with discrete PCM islands on top, optically connecting the presynaptic (pre‐neuron) and the postsynaptic (post‐neuron) signals. The red open circle is a circulator with port 2 and port 3 connecting the synapse and the post‐neuron; weighting pulses are applied through port 1 to the synapse. (c) Top: Schematic shows photonic synapse with a standard design: a straight waveguide with a thin film of GST (6 µm × 0.8 µm, orange block) on top. Bottom: TE mode *E*‐field distribution at the surface of the waveguide with the entire GST film (white box) in the crystalline state. (d) Top: Schematic shows photonic synapse with synapse‐mimic design: a tapered waveguide with six discrete GST islands (1 µm × 0.8 µm each) on top, which is the structure used in the experiments. Bottom: *E*‐field distribution with all GST islands in crystalline states. (e) *E*‐field distributions along the center line of the waveguide surface. The yellow, purple, green, and cyan curves correspond to the *E*‐field distribution along the dashed horizontal lines in (c) and (d) (standard and synapse‐mimic). The dashed red (black) lines illustrate the left and right boundaries of the GST film (discrete GST islands) in standard designs. (f) Top: Statistical results of the *E*‐field inside the GST film or islands (square, average value; box, SD; bottom and top lines, minimum and maximum values). Bottom: The ratio between the average *E*‐field at the output and input edges of the GST film or islands corresponding to the orange dashed lines in (c) and (d). Reproduced with permission [110]. Copyright 2017, Science.

In transparent photonic neuromorphic devices, memory and synaptic weighting are realized using optical materials, as illustrated in Figure 5a,b. Phase‐change chalcogenide materials provide another avenue, especially in the infrared, though several compounds are transparent into the visible. Classical PCMs like Ge_2_Sb_2_Te_5_ (GST) are opaque in visible light (bandgap ≈ 0.7 eV). For instance, Cheng et al. deposited patterned GST islands on a silicon‐nitride waveguide to form a photonic synapse [110]. An optical pulse raises the GST temperature, switching part of it from crystalline to amorphous. This changes the waveguide's transmittance, thereby adjusting the “synaptic weight”. Using paired pre‐ and post‐synaptic pulses and an interferometer, they reproduced spike‐timing‐dependent plasticity (STDP), where the weight change depended on the relative timing of spikes. Similar designs use GST on microring resonators: by placing GST on dual parallel rings, a passive “integrate‐and‐fire” photonic neuron was realized. The incident light plays a critical role in the process by effectively “writing” weight into the PCM through a heating mechanism. This alteration in the PCM's state facilitates the modulation of its properties, allowing it to store information akin to how synaptic weights function in biological neurons. Subsequently, the interference patterns generated by the signals travelling through the system, specifically between the through‐port and drop‐port, allow us to “read” the membrane potential of the neuron. This interplay between light and signal interference is essential for accurately interpreting the neuron's activity, thereby mimicking neural communication in a bio‐inspired computational framework. Schematics in Figure 5c and d show the photonic synapse with a standard design and the photonic synapse with a synapse‐mimic design, respectively. Figure 5e and f show the electric‐field distributions along the center line of the waveguide surface and the statistical results of the electric‐field inside the GST film or islands. Alloys that contain reduced amounts of tellurium (Te) tend to exhibit considerably larger band gaps, which can significantly impact their electrical and optical properties. For instance, Ge‐Sb‐Se‐Te alloys (GSST) and binary Sb_2_S_3_/Sb_2_Se_3_ have band gaps on the order of 1–2 eV [111]. Sb_2_S_3_ (Eg ≈ 1.8 eV) and Sb_2_Se_3_ (Eg ≈ 1.2 eV) can be nearly transparent at red/near‐IR wavelengths, and have been demonstrated in low‐loss photonic memory circuits [111]. These materials can be optically or electrically switched between amorphous and crystalline states, with large refractive‐index contrast but relatively small absorption in the visible band. In neuromorphic contexts, Sb_2_S_3_‐based devices show persistent photoconductivity and step‐like (nonvolatile) conductance changes under light pulses, enabling long‐term synaptic storage [111]. Similarly, Sb_2_Se_3_, with a slightly smaller bandgap, supports low‐energy IR switching while still allowing some visible transparency [111]. Other emerging PCMs include Ge‐Sb‐Se_5_, In‐Se compounds, and GST derivatives; the trend is to increase bandgap via Se/S substitution to improve transparency while retaining fast switching.

Building on these concepts, Feldmann et al. demonstrated an all‐optical neural network with 4 photonic neurons and 60 PCM synapses on‐chip [112]. The system was designed to precisely direct optical pulses to each weight using a microring wavelength demultiplexer, allowing for enhanced control and modulation of the synaptic signals. This architecture not only enhances the network's functionality but also facilitates intricate data processing by enabling multiple wavelengths to be utilized simultaneously, thereby increasing the overall bandwidth and efficiency of the synaptic connections. The network learned with supervised and unsupervised light signals and successfully recognized optical patterns. In addition to PCMs, other photonic memory elements have been explored.

2D materials create exceptionally thin channels that allow for enhanced interactions between light and matter. These materials feature surfaces that are free from dangling bonds, which helps to improve their stability and performance in various applications [113]. For example, graphene (a zero‐bandgap semi‐metal, ≈ 97.7% transmittance per monolayer) acts as an ultrafast, transparent conductor and photodetector; when coupled to photoactive quantum dots (e.g. perovskite QDs), it yields devices with enormous responsivity (≈ 1.4 × 10^8^ A/W) and light‐assisted memory [114]. Similarly, TMDs like MoS_2_ (bandgap ≈ 1.2–1.8 eV) have strong visible absorption; when integrated with a wide‐gap oxide (e.g. ZnO, Eg ≈ 3.3 eV), the heterostructure exhibits persistent photoconductivity and multi‐wavelength synaptic responses [115]. Lead‐free 2D halide perovskites such as phenethylammonium tin iodide (PEA_2_SnI_4_) combine moderate visible‐bandgap (≈ 1.8–2.0 eV) absorption with long‐lived trap‐mediated photocurrents. Indeed, PEA_2_SnI_4_ devices have demonstrated photonic short‐term and long‐term plasticity (pulse‐integrating EPSC) with ≈ 60 A/W responsivity at 650 nm [116]. More generally, 2D semiconductors (e.g., WS_2_, In_2_Se_3_, etc.) are being explored as transparent synaptic layers in phototransistors and memristors [113, 115].

**FIGURE 6:**
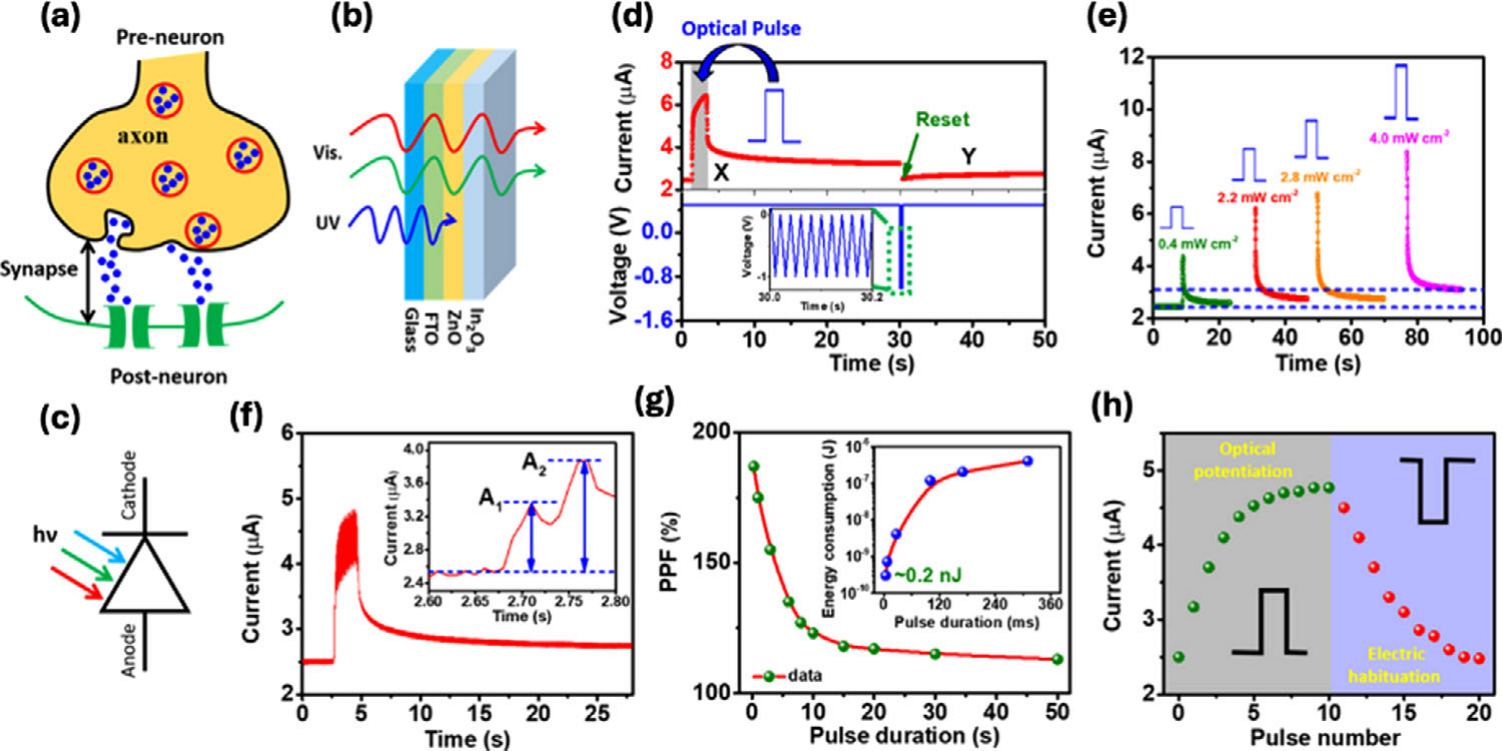
(a, b) Schematics of a synapse structure within the human brain, and our two‐terminal visible light transparent device, respectively. (c) Sketch of a photodetector, which is very similar to the artificial synapse. (d) Top panel shows the transient responses of the device after illumination with a single optical pulse (intensity: 0.4 mW cm^−2^, duration: 2 s, marked by the gray colored area), showing a gradual decay after the UV is switched off (point X). The bottom panel depicts the applied measuring voltage (0.6 V) and pulses of −1 V after 30 s; the inset shows the shape of the applied electric pulses. (e) Transient current–time characteristics for four optical pulses with different intensities from 0.4 to 4 mW cm^−2^, showing the dynamic behavior of current with the increasing incident light intensity of a single pulse. (f) Change in the current under the action of applied sequential optical pulses. The inset shows the change in current amplitude for two sequential optical pulses. (g) Change in the PPF index with different optical frequencies. The inset shows the energy consumed per synaptic event as a function of optical pulse duration. (h) Gradual current change across the synaptic devices after applying a train of photonic pulses (intensity: 0.4 mW cm^−2^, duration and separation: 1 s) and negative electrical pulses (−1 V, duration and separation: 20 ms). Top and bottom insets show the shape of the applied pulses. Reproduced with permission [103]. Copyright 2018, American Chemical Society.

In the context of photonic‐enabled transparent neuromorphic devices, transparent conductive oxides (TCOs) and perovskite oxides emerge as essential materials, offering unique electrical and optical characteristics that enhance the functionality and efficiency of these advanced technologies. TCOs facilitate efficient light management while supporting electrical conduction, making them ideal for integrating with photonic circuits. Simultaneously, perovskite oxides contribute to the adaptive processing capabilities of neuromorphic systems, paving the way for innovative solutions in computing and information processing. TCOs such as indium tin oxide (ITO), ZnO, Al‐doped ZnO (AZO), or indium–gallium–zinc oxide (IGZO) have wide band gaps (>3.0 eV) and excellent visible transparency. These oxide layers can be integrated on silicon or glass waveguides to realize all‐optical synaptic elements. Recent work argues that TCOs inherently exhibit optical nonlinearity and bistability, making them ideal platforms for photonic neural networks [117]. For example, ITO or In_2_O_3_ can serve as transparent electrodes or active layers in photonic memristors. Kumar et al. reported a transparent photonic synapse made of all‐oxide thin films (In_2_O_3_/ZnO) on glass, as schematically shown in Figure 6a and b [103]. Under UV illumination, the device's conductivity exhibited synaptic plasticity—STP, LTP, PPF, etc. The synapse is optically transparent and CMOS‐compatible, demonstrating low‐cost photonic synaptic behavior. Figure 6c illustrates a sketch of a photodetector, which is very similar to an artificial synapse. Figure 6d and e show the transient responses of the In_2_O_3_/ZnO‐based device after illumination with a single optical pulse (intensity: 0.4 mW cm^−2^, duration: 2 s, marked by the gray colored area) and transient current–time characteristics for four optical pulses with different intensities from 0.4 to 4 mW cm^−2^, showing the dynamic behavior of current with the increasing incident light intensity of a single pulse. Figure 6f,g show the change in the current under the action of applied sequential optical pulses and the change in the PPF index with different optical frequencies. Figure 6h exhibits the gradual current change across the synaptic devices after applying a train of photonic pulses (intensity: 0.4 mW cm^−2^, duration and separation: 1 s) and negative electrical pulses (−1 V, duration and separation: 20 ms).

Perovskite oxides like barium strontium titanate (Ba_0_._5_Sr_0_._5_TiO_3_, Eg ≈ 3.2 eV) combine wide‐gap transparency with ferroelectric‐like switching. Shrivastava et al. reported BaSrTiO_3_‐based photomemristor (with ITO/AZO electrodes) demonstrated analog resistive switching and both photo‐excitation and photo‐inhibition (LTP/LTD) under 405 and 633 nm illumination [118]. In summary, transparent oxide semiconductors offer an efficient solution for low‐loss optical transmission, making them ideal for various advanced technologies. These materials can be specifically tailored through methods such as doping, which involves the introduction of impurities, or by controlling oxygen vacancies within their structure. This engineering flexibility allows these semiconductors to exhibit synaptic plasticity, a vital property for mimicking the behavior of biological synapses. As a result, they can operate at high speeds and low voltages, enhancing the performance and energy efficiency of electronic systems [117, 118].

**FIGURE 7:**
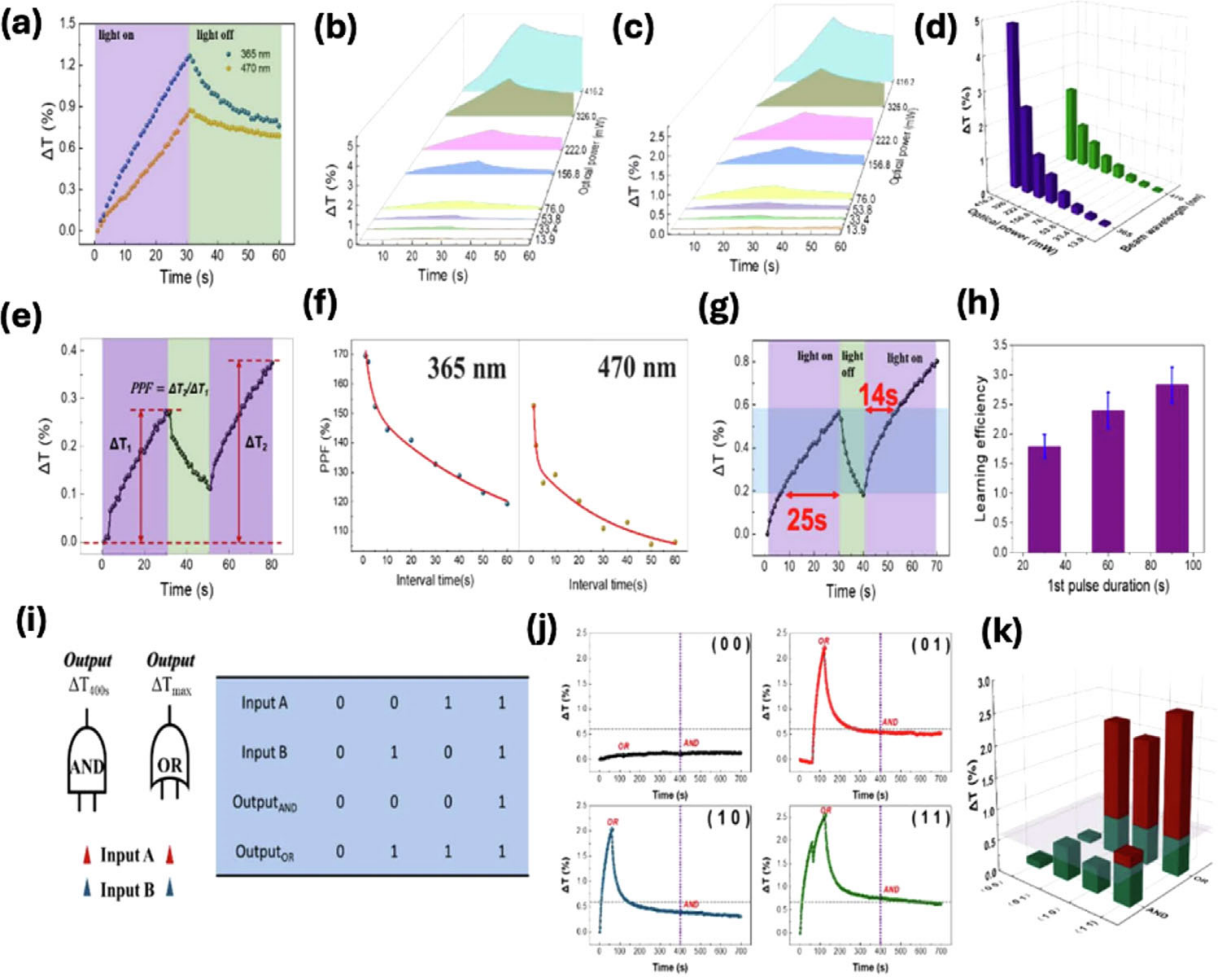
(a) Changes of transmittance at 600 nm under 222 mW LED irradiation. Transmittance change curves under 365 (b) and 470 nm (c) LED irradiations with various durations. (d) The relationship between maximum transmittance changes and LED powers. (e) PPF behaviors under 76 mW 365 nm beam irradiation. (f) The relationship of the PPF indexes and the intervals between 76 mW 365 (left) and 470 (right) nm LED pulses. (g) The mimicking of learning ability under 156.8 mW 365 nm LED illumination. (h) Learning efficiencies with different durations of the first pulses. (i) Illustration of the logical processing “AND” and “OR” on the all‐photonic synapse. (j) Transmittance change curves for logical information processing. (k) Transmittance change values demonstrating the logical processing. Reproduced with permission [120]. Copyright 2024, Nature.

Research is increasingly focused on hybrid organic/inorganic materials and colloidal nanostructures due to their promising properties. Organic semiconductors, which consist of π‐conjugated polymers and small molecules, stand out for their exceptional transparency outside of their absorption bands, making them ideal for applications where clear visibility is essential. Additionally, these materials are relatively easy to process, allowing for versatile manufacturing techniques. However, it's important to note that organic semiconductors generally have slower switching speeds compared to their inorganic counterparts, which can limit their performance in certain applications. Bulk‐heterojunction systems have shown broadband photonic synaptic behavior with low drive voltage [119]. Of particular interest are colloidal quantum dots (QDs) and perovskite nanocrystals. Metal‐halide perovskite QDs (e.g. CsPbX_3_) have tunable band gaps (≈ 1.5–3 eV) and extremely high photoresponsivity. For instance, graphene‐perovskite‐QD heterostructures achieved responsivities ≈ 10^8^ A/W and clear light‐induced memory in an ultrathin synaptic transistor [114]. In general, 0D/2D hybrids (graphene/PQDs, MoS_2_/PQDs, etc.) leverage the transparency of the conductor (graphene or TiO_2_) with the absorption of the QDs to yield strong optical gain and synaptic plasticity. Hybrid organic‐inorganic perovskites likewise combine high absorption with potential transparency at certain wavelengths. The lead‐free 2D perovskite compound PEA_2_SnI_4_ is capable of converting visible light into a sustained photocurrent that exhibits a slow decay over time. This behavior closely resembles the dynamics of an excitatory postsynaptic current, which is a critical mechanism in neural signaling. Additionally, PEA_2_SnI_4_ demonstrates a cumulative response to pulse trains, indicating its potential for applications in optoelectronic devices and photonic systems [116]. More recently, all‐photonic memories using photochromic perovskites were shown: a CsPbIBr_2_ film undergoes a reversible structural change under UV/visible light, altering its optical transmittance as shown in Figure 7a–d [120]. The induced transmittance change persists after the light is removed, enabling “light‐driven optical memory” without any electrodes. Such perovskite synapses achieved full‐bit accuracy on simple image tasks with zero in‐situ energy (no electrical readout) as illustrated in Figure 7e–k. In 2020 Feldmann et al. used a chip‐based optical frequency comb and a bank of PCM cells as a “photonic tensor core,” achieving 10^12^ MAC (multiply‐accumulate) operations per second [121]. These results confirm the potential of integrated photonic memories for ultrafast neuromorphic computation.

### 3.2 Photonic Neurons

Photonic neurons are optical counterparts of biological spiking neurons that perform signal integration and threshold‐triggered firing using light as the computational medium, relying on intrinsic optical nonlinearities, such as gain saturation, bistability, or threshold switching, to accumulate inputs and generate ultrafast spikes. The development of photonic neurons has progressed from early optical analog computing to fully integrated neuromorphic photonic platforms. More recently, the realization of spiking photonic neurons using phase‐change–assisted resonators and excitable laser cavities has established the foundational device physics required for ultrafast, energy‐efficient neuromorphic photonic systems.

Realizing a high‐performance photonic neuron device requires the co‐optimization of device architecture and material functionality to support analog integration, nonlinear activation, rapid spiking, and large‐scale on‐chip integration. Structurally, waveguide‐based neurons on Si/SiN platforms employ interferometric meshes, microring resonators, and photodetector–modulator pairs to perform weighted summation and reconfigurable nonlinear transformation with CMOS compatibility and low propagation loss. Laser‐based excitable neurons, such as VCSEL‐SA and FP‐SA (Fabry‐Perot saturable absorber) cavities, use gain‐absorption dynamics to generate GHz‐scale optical spikes, enabling ultrafast neuromorphic processing. Yan et al. reported a complete photonic integrated neuron (PIN) on SiN, where MRRs supply both Kerr‐based nonlinear activation and reconfigurable temporal delay, enabling multi‐kernel convolution and sub‐nanosecond spatiotemporal processing. This work shows that nonlinearity completeness and programmable temporal convolution are emerging as central requirements for scalable all‐optical neurons [122].

**FIGURE 8:**
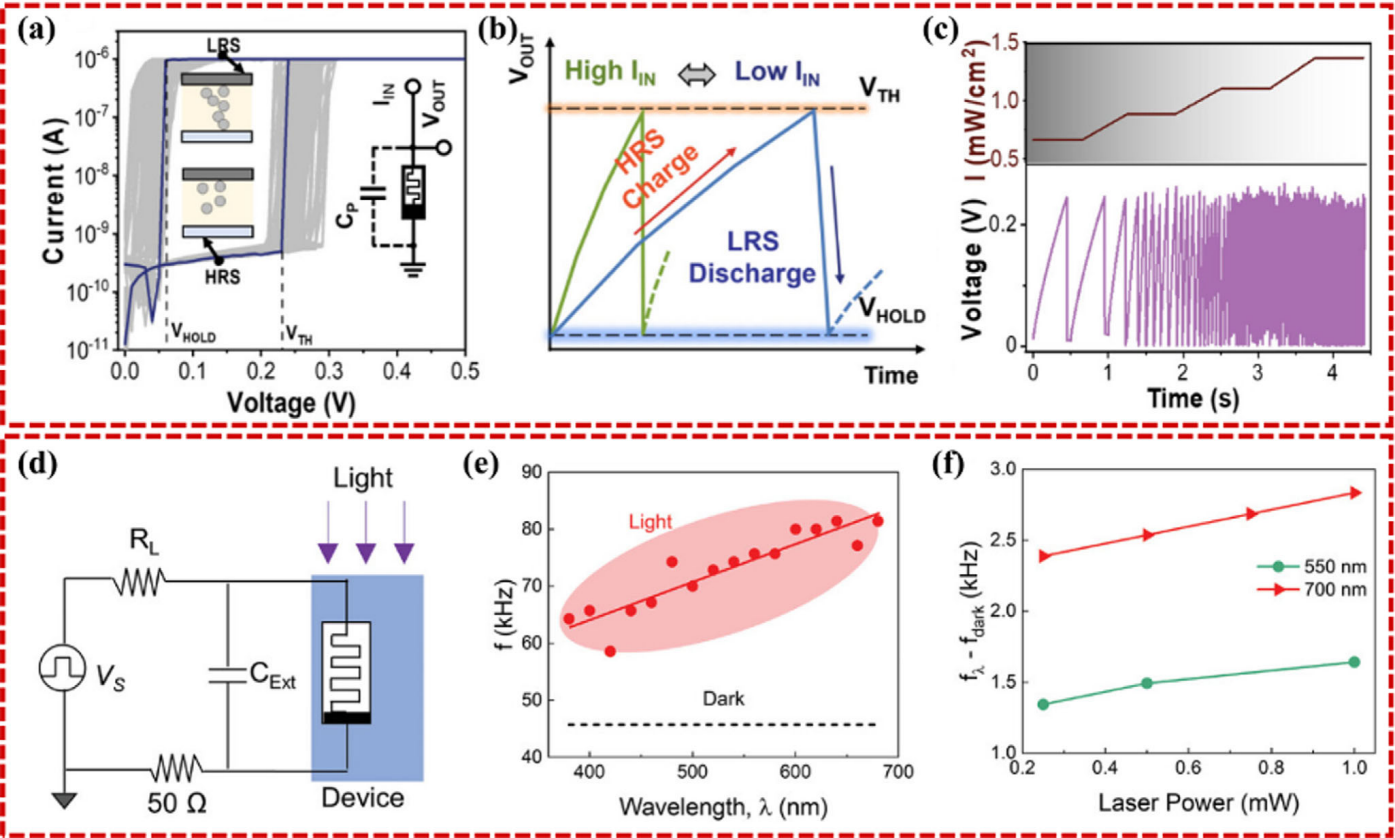
The optoelectrical characteristics of the TaO_X_‐based memristors and its neuromorphic functions. (a) *I*‐*V* characteristics curves of the memristors under 200 consecutive cycles. (b) The spike coding mechanism of the artificial neuron. (c) Dependence of the spiking encoding frequency on incident light intensity in the device. Reproduced with permission [123]. Copyright 2025, Wiley‐VCH. The firing behavior of VO_X_ based LIF neurons under different light stimuli. (d) Schematic diagram of the circuit employed to characterize the oscillation dynamics. (e) Dependence of the oscillation frequency on the excitation wavelength. (f) Modulation of oscillation frequency by incident light intensity. Reproduced with permission [124]. Copyright 2024, Wiley‐VCH.

However, constrained by energy consumption and functional requirements, traditional photonic neuron structures have gradually failed to meet the demands of development. Memristor‐based photonic neurons surpass traditional photonic neuron structures by intrinsically combining integration, nonlinear activation, memory, and optical modulation within a single nanoscale threshold‐switching element, enabling far greater compactness, energy efficiency, and functional density than resonator‐ or interferometer‐based architectures. Memristor–capacitor oscillatory neurons exploit volatile threshold switching to emulate LIF dynamics. In this structure, traditional metal oxides provide volatile threshold switching and insulator‐metal transition (IMT)‐driven oscillations, critical for LIF spiking. Hybrid transistor–memristor neurons, as shown by Qiao et al., integrate a photochromic film, an IGCdO phototransistor, and a TaO_X_ memristor to create a pupillary reflex–inspired adaptive spiking visual neuron with a 160‐dB dynamic range [123]. Phototransistors rely on light‐modulated carrier generation within semiconductor channels, enabling tunable gain and fast excitability, whereas photomemristor neurons exploit volatile threshold switching to emulate LIF dynamics with compact, passive device structures. The TaO_X_ memristor *I*‐*V* curves demonstrate that the memristor exhibits stable and reliable threshold resistive switching behavior (Figure 8a). Due to the high dielectric constant and appropriate thickness of the TaO_X_ layer, a significant parasitic capacitance arises. This capacitance serves as an essential prerequisite, enabling a single TaO_X_‐based memristor to encode input current pulses into output pulse signals (Figure 8b). Figure 8c shows the dynamic response of spiking encoding with increased light intensity. The 1T1M structure vertically integrated with an IGCdO‐based transistor and a TaO_X_ ‐based memristor possesses the conversion of applied light intensity into spike signals. This hybrid structure highlights a key design philosophy: decoupling optical adaptation, photodetection, and spike generation into modular functional layers. Beyond conventional oxide materials, Phase‐change materials enable nonvolatile weighting and thresholding. Nath et al. investigated optically tunable electrical oscillations in a vanadium oxide–based memristor (V_3_O_5_) and proposed an electroforming‐free neuronal oscillator [124]. This study designed a circuit to measure oscillatory dynamics (Figure 8d) and demonstrated that oscillation frequency can be determined by the light wavelength and incident light intensity (Figure 8e,f), enabling direct light‐controlled LIF dynamics, which further demonstrated in‐sensor reservoir computing and an optical encoding layer for SNNs, although noise robustness and long‐term stability remain limiting factors.

### 3.3 All‐Optical Logic in Photonics

Optical circuits are powerful technologies that can perform logic functions such as AND, OR, and XOR at the speed of light, offering significant advantages in terms of speed and efficiency compared to traditional electronic circuits. A key component in these circuits is the MZI, which uses two beam splitters and two paths to manipulate light. When integrated with nonlinear elements, such as semiconductor optical amplifiers, nonlinear fibers, or PCMs, these interferometers can act as all‐optical gates. In this context, the interferometers work by allowing light pulses to travel through different paths and then recombine, creating interference patterns. The resulting logic outputs are determined by the phases or intensities of the input light pulses. By carefully controlling these factors, the optical circuits can produce desired logic states, enabling faster and more efficient processing of information. This technology holds great potential for future advancements in optical computing and communication systems, making them critical in the development of high‐speed data transfer and processing solutions. Photonic crystals and metasurfaces have also been proposed for compact logic gates. In general, photonics offers a wealth of linear and nonlinear interactions that can realize logic operations with minimal crosstalk and massive parallelism. For example, a single microring with Kerr nonlinearity can behave as an optical NAND gate at gigahertz rates. While fully optical logic systems have not yet reached the level of sophistication found in photonic neural units, there are a variety of innovative designs available that can effectively route and blend optical spikes. These technologies facilitate all‐optical signal processing, eliminating the need for electronic conversion and paving the way for faster, more efficient communication systems.

### 3.4 Hybrid Electronic‐Photonic Architectures

While “transparent” photonic devices primarily concentrate on the flow of optical information, real‐world applications frequently involve a hybrid approach that combines both optical and electronic elements. Integrated photonic chips, essential for modern communication and computing technologies, often rely on electrical control mechanisms to operate key components such as phase shifters, which manipulate light paths; detectors, which convert optical signals into electrical signals; and sources, which generate light. This integration enhances the functionality and performance of photonic systems, enabling more efficient data processing and transmission in various applications, including telecommunications and sensing technologies. For instance, silicon photonic neural networks use thermo‐optic or electro‐optic phase shifters to adjust weights, and photodetectors to read outputs, making them partially hybrid.

To maximize performance, researchers combine diverse materials in hybrid photonic integrated circuits (PICs). Graphene or 2D layers on top of silicon waveguides yield high‐speed electro‐absorption modulators, merging compact optics with electrical gating. Strong Pockels materials (LiNbO_3_, BaTiO_3_) and III‐V gain sections have been integrated on silicon to add functionality (fast phase shifters, on‐chip lasers, detectors). Hybrid “electro‐optic” accelerators, for example, embed photonic analog MAC cores alongside analog electronic charge accumulation. Such systems “complement analog photonic computation units with analog electronic processes to improve scalability”, effectively extending the size of optical matrix operations by using electronic memory [125]. Table 2 summarizes representative transparent (fully optical) vs. hybrid photonic neuromorphic architectures. Transparent photonic neurons and PCM synapses operate in the optical domain for ultralow latency, whereas practical systems often incorporate electronics for stability, programmability, or readout. The field continues to advance toward more integrated, fully photonic neuromorphic chips; however, current architectures often combine optics with CMOS control.

**TABLE 2:** Summarizes representative transparent (fully optical) vs. hybrid photonic neuromorphic architectures.

Architecture	Components & Behavior	Transparency/Hybrid
DFB–SA photonic neuron	DFB laser + saturable absorber; excitable spikes on optical input	Fully optical neuron; no electronics in the core
VCSEL‐based SNN	VCSEL lasers + delay loops; temporal multiplexing of inputs; ultrafast optical spikes	Fully optical spiking network (all processing in light)
Silicon MZI photonic network	Si waveguide mesh + thermo‐/electro‐optic phase shifters + PDs	Hybrid: photonic MAC core, but requires electronic drivers/heaters and photodetection
PCM synaptic array	Waveguides or rings with GST cells; optical pulses program weights	Fully optical weight storage; programmable by light pulses
Transparent oxide synapse	In_2_O_3_/ZnO photoconductive films on glass; UV‐driven plasticity	Fully optical (transparent substrate); electronic current output but photo‐driven
Graphene–Si modulators	Si waveguide + graphene layer; electrical gate tunes absorption	Hybrid: optical transmission with electronic voltage control

## 4 Applications and Future Directions

In summary, PICs showcase remarkable strengths in energy efficiency, enabling them to consume less power while performing various tasks. They excel in parallel processing, allowing multiple operations to occur simultaneously, which enhances their overall speed and performance. However, despite these advantages, PICs significantly lag behind electronic integrated circuits (ICs) in several critical areas. Most notably, they are generally more expensive to produce, making them less accessible for widespread use. PICs face significant challenges when it comes to scaling up for larger systems. These circuits can be quite complex to integrate with current technologies, often requiring specialized knowledge and equipment. Furthermore, their larger physical size can restrict their use in environments where space is valuable, such as in compact electronic devices or within crowded data centers. Interestingly, many of the advanced PICs available today resemble the ICs that were first manufactured by Intel nearly 60 years ago, highlighting the ongoing struggle to advance these technologies further. As a consequence, we remain several decades from witnessing the integration of photonic chips into everyday devices like smartphones, laptops, and iPads. The road ahead is even longer for the development of advanced photonic computers, which will possess the capability to execute large‐scale AI models, such as ChatGPT and AlphaGo. These cutting‐edge machines would also be equipped to tackle intricate scientific challenges, including molecular simulations that explore the behavior of particles at the atomic level, and finite‐element analyses that help engineers design and evaluate complex structures.

In this context, it is essential to emphasize the remarkable capabilities of the transparent (Al,Ga)N/GaN nanowire array, which has been ingeniously integrated into a dual‐mode photonic synaptic device. These advanced nanowire films exhibit exceptional transparency in the visible light spectrum, allowing for seamless integration into various optical applications. They possess the unique ability to switch between two distinct modes: fast photodetection at a neutral 0 V bias and programmable synaptic behavior when an external bias is applied. As a result, these nanowires facilitate efficient on‐chip vision processing, achieving this with remarkably low energy consumption. For example, each synaptic event operates with an astonishingly low energy cost of approximately 2.5 × 10^−^^14^ J, making them highly efficient for next‐generation bio‐inspired computing applications [126]. The integration of transparency and neuromorphic functionality positions these technologies as highly suitable for edge‐AI applications within smart displays and sensors.

### 4.1 Edge AI and In‐Sensor Computing

Photonic neuromorphic circuits excel in speed and energy efficiency for edge computing. They perform parallel processing of optical inputs directly at the sensor (in‐sensor computing), greatly reducing data movement [127]. Integrated photonic neural networks using microring weight banks and broadcast‐and‐weight architectures can achieve high bandwidth inference. For example, a photonic “Netcast” system demonstrated ≈ 99% image classification accuracy using only ≈ 10 aJ per multiply‐accumulate operation [128]. Such extremely low energy per operation and high throughput make photonic neuromorphic devices ideal for always‐on on‐device AI without cloud dependence [129].

### 4.2 Transparent Displays

Neuromorphic devices can be embedded in transparent displays and smart windows. TCOs like IGZO offer >80% visible transparency and have been used in optical synaptic transistors via their persistent photoconductivity [130]. By integrating transparent photodiodes with photonic synapses, one can build see‐through “sensory displays” that capture and process images simultaneously. For instance, dual‐mode GaN nanowire devices enable 360° photodetection and synaptic memory in a single transparent unit [126]. Innovative transparent neuromorphic surfaces have the potential to seamlessly perform edge detection and object recognition in augmented reality (AR) and virtual reality (VR) applications directly on the glass. This cutting‐edge technology could enable users to interact with digital elements overlaid onto their real‐world environment, enhancing the immersive experience and facilitating intuitive interactions.

### 4.3 Wearables and Flexible Systems

Photonic neuromorphic hardware is also promising for wearables and robotics. Flexible, printed neuromorphic arrays have been demonstrated (e.g., printed artificial synapses that monitor and process multimodal physiological signals for health diagnostics) [131]. These chip‐less systems integrate sensing and processing in a conformal form factor, enabling applications like real‐time biomarker monitoring or smart prosthetics. The development of transparent photonic synapses embedded in flexible substrates has the potential to revolutionize technology, paving the way for advanced adaptive heads‐up displays and innovative soft contact lenses. These cutting‐edge devices could seamlessly process visual and biometric data while consuming minimal power, creating a new standard for efficiency and portability in wearable technology. Imagine contact lenses that not only provide vision correction but also deliver real‐time health monitoring and information directly to the wearer, all without the bulk and power demands of traditional devices.

### 4.4 Scalability and Integration

Scaling neuromorphic photonic systems to large networks requires novel architectures and materials. Techniques like WDM with microring weight banks allow many channels on a single chip [129]. Broadcast loops and wavelength‐switched photonic networks have been proposed as innovative solutions to interconnect a vast number of neurons. These systems utilize fixed optical resources to facilitate high‐speed communication between neurons, enabling efficient data transfer and integration within complex neural architectures. By leveraging these advanced networking technologies, researchers aim to enhance the connectivity and functionality of neural networks, ultimately advancing our understanding of brain‐like processing and computational efficiency. Equally important is heterogeneous integration: co‐packaging photonic chips with electronics and nonvolatile memory (e.g., Ps) can overcome interconnect bottlenecks [132]. Future devices will need to incorporate multiple materials (III‐V semiconductors, silicon photonics, 2D crystals, chalcogenides, etc.) and leverage 3D chiplet packaging to achieve the neuron densities required for practical AI.

### 4.5 Energy Efficiency and Materials

Photonic neuromorphic devices already achieve brain‐like efficiency by avoiding costly data movement. They operate in the femtojoule (or even attojoule) regime per operation [128]. Further gains are expected by optimizing device designs (e.g. high‐Q resonators, low‐loss waveguides) and materials. For example, integrating photonic phase‐change films can provide nonvolatile weight storage with optical programmability. Novel transparent materials and device concepts, such as 2D/organic photonic components or metasurface‐assisted filters, will enable compact, low‐voltage operation. Tackling the complexities associated with achieving uniform fabrication, ensuring material stability, and effectively managing thermal dynamics is essential for the successful development of energy‐efficient transparent neuromorphic platforms. These advancements will enable the creation of more reliable and high‐performance systems that can revolutionize the field of artificial intelligence and adaptive computing.

### 4.6 Neuromorphic Bionics

Photonic neuromorphic devices provide a powerful platform for realizing biologically inspired neural systems by closely mimicking the sensing, processing, and adaptation mechanisms of natural nervous systems. By integrating photonic neurons and synapses with in‐sensor computing architectures, these devices can directly process sensory information, such as visual, optical, or physiological signals, at the point of acquisition, analogous to biological retinas and peripheral neural circuits. Their inherent advantages in ultrahigh bandwidth, massive parallelism, and ultralow energy consumption enable real‐time spatiotemporal processing, spike‐based encoding, and adaptive learning behaviors that are difficult to achieve with conventional electronics. When implemented in transparent, flexible, or wearable form factors, photonic neuromorphic systems further enable bionic interfaces such as artificial retinas, sensory skins, and adaptive human–machine interfaces, seamlessly coupling perception and computation. Together with scalable photonic network architectures and heterogeneous material integration, these bio‐inspired photonic platforms make it possible to translate neuromorphic principles into compact hardware that operates under realistic constraints of power and latency.

## 5 Conclusions

This review provides an in‐depth examination of the latest advancements in transparent neuromorphic photonic systems, emphasizing their significance within the realms of photonic engineering and materials science. It critically assesses both the emerging challenges that researchers face and the anticipated obstacles that may arise in the near future. Additionally, the review outlines the essential scientific principles and technological innovations required to address these challenges effectively. By mapping out potential solutions and innovations, this analysis aims to guide future research efforts and facilitate the development of more efficient and capable transparent neuromorphic photonic systems. Photonic‐based transparent neuromorphic devices represent a promising approach for next‐generation edge computing. By combining optics with brain‐inspired circuits, these systems achieve significant improvements in both bandwidth and energy efficiency. Their unique transparency and flexibility enable the creation of entirely new form factors, such as see‐through sensors, displays, and wearables that incorporate embedded intelligence.

Continued innovation in materials, like transparent oxides, PCMs, and 2D materials, will be essential, along with advancements in device architectures, which include all‐optical logic in photonics, photonic memory and synapses, hybrid electronic‐photonic architectures, and dual‐mode sensors. Additionally, heterogeneous integration, such as co‐packaged photonics with memory, will play a crucial role in scaling these systems. In the future, innovative transparent neuromorphic platforms are poised to revolutionize a diverse array of applications. Imagine AR and VR glasses that seamlessly process visuals in real‐time, enhancing our immersive experiences with clarity and precision. Additionally, think of advanced smart health patches that monitor vital signs and deliver instant feedback, along with sophisticated robotic skins that mimic human touch and respond intuitively to their environment. These breakthroughs will enable autonomous, low‐power artificial intelligence to operate efficiently at the edge, transforming how we interact with technology and the world around us. These advancements highlight a significant convergence of display and computing technologies, leading to the development of sophisticated vision systems. These systems possess the remarkable ability to “see” their surroundings and learn from their experiences with an efficiency reminiscent of the human brain. This integration promises to enhance the way machines interact with the world, facilitating a deeper understanding and interpretation of visual data.

## Author Contributions

**Hangfei Li**: writing – review and editing, formal analysis. **Ye Zhou**: writing – review and editing, validation, formal analysis. **Yu Wen**: writing – review and editing, formal analysis. **Junghyeon Lee**: writing – review and editing, formal analysis. **Seunghee Cho**: writing – review and editing, formal analysis. **Malkeshkumar Patel**: writing – review and editing, formal analysis. **Ki‐bum Lee**: writing – review and editing, formal analysis. **Joondong Kim**: writing – review and editing, funding acquisition, project administration, supervision, resources, validation, conceptualization, formal analysis. **Shuvaraj Ghosh**: writing – original draft, conceptualization, visualization, formal analysis, writing – review and editing.

## Conflicts of Interest

The authors declare no conflicts of interest.

## Data Availability

The data that support the findings of this study are available from the corresponding author upon reasonable request.
